# Cytokinin perception in potato: new features of canonical players

**DOI:** 10.1093/jxb/ery199

**Published:** 2018-05-24

**Authors:** Sergey N Lomin, Yulia A Myakushina, Oksana O Kolachevskaya, Irina A Getman, Dmitry V Arkhipov, Ekaterina M Savelieva, Dmitry I Osolodkin, Georgy A Romanov

**Affiliations:** 1 Timiryazev Institute of Plant Physiology, Russian Academy of Sciences, Moscow, Russia; 2 Institute of Poliomyelitis and Viral Encephalitides, FSBSI Chumakov FSC R&D IBP RAS, Poselok Instituta Poliomelita 8 bd 1, Poselenie Moskovsky, Moscow, Russia; 3 Sechenov First Moscow State Medical University, Moscow, Russia; 4 Belozersky Institute of Physico-Chemical Biology, Lomonosov Moscow State University, Leninskie Gory, Moscow, Russia

**Keywords:** CHASE domain-containing histidine kinase, cytokinin, cytokinin receptor, cytokinin signaling, gene expression, hormone perception, potato, *Solanum tuberosum*

## Abstract

Potato is the most economically important non-cereal food crop. Tuber formation in potato is regulated by phytohormones, cytokinins (CKs) in particular. The present work studied CK signal perception in potato. The sequenced potato genome of doubled monoploid Phureja was used for bioinformatic analysis and as a tool for identification of putative CK receptors from autotetraploid potato cv. Désirée. All basic elements of multistep phosphorelay required for CK signal transduction were identified in the Phureja genome, including three genes orthologous to three CK receptor genes (*AHK 2–4*) of Arabidopsis. As distinct from Phureja, autotetraploid potato contains at least two allelic isoforms of each receptor type. Putative receptor genes from Désirée plants were cloned, sequenced and expressed, and the main characteristics of encoded proteins were determined, in particular their consensus motifs, modelled structure, ligand-binding properties, and ability to transmit CK signals. In all studied aspects the predicted sensor histidine kinases met the requirements for genuine CK receptors. Expression of potato CK receptors was found to be organ-specific and sensitive to growth conditions, particularly to sucrose content. Our results provide a solid basis for further in-depth study of CK signaling system and biotechnological improvement of potato.

## Teaser

Potato genes for putative cytokinin receptors were cloned and expressed. The proteins displayed properties of cytokinin receptors, and their expression *in planta* was organ specific and sensitive to growth conditions and sucrose content.

## Introduction

Potato is a widespread and important crop whose tuber formation is controlled by phytohormones (reviewed in Aksenova et al., 2012, 2014). Previous studies have shown that cytokinins (CKs) and auxins can accelerate and enhance potato tuber formation (Aksenova *et al.*, 2000; Romanov *et al.*, 2000; Roumeliotis *et al.*, 2012; Kolachevskaya *et al.*, 2015, 2017; Wang *et al.*, 2018). In non-tuberizing plants (tobacco, tomato), increased doses of active CKs stimulate morphogenesis, in many aspects resembling tuber formation (Guivarc’h *et al.*, 2002; Eviatar-Ribak *et al.*, 2013). CK signaling is also involved in the formation of nodules on the roots of legumes (reviewed in Frugier *et al.*, 2008; Miri *et al.*, 2016). CKs largely determine the nature of source–sink relationships in the whole plant, enhancing the attracting ability of the tubers (Abelenda and Prat, 2013). Elevated doses of CKs affect the overall architectonics of potato plants, suppressing root development (Aksenova *et al.*, 2000). In addition, CKs participate in plant defense against biotic and abiotic adverse factors (Zwack and Rashotte, 2015; Brütting *et al.*, 2017; Thu *et al.*, 2017). All the above indicates the important role of CKs in both the formation of tubers and the general development and resistance of potato plants.

The molecular mechanism of CK action on a plant cell has been established using mainly the Arabidopsis model (reviewed in Hutchison and Kieber, 2002, Hwang *et al.*, 2002; Kakimoto, 2003; Heyl and Schmülling, 2003; Sakakibara, 2006; Müller and Sheen, 2007). This mechanism is based on multistep phosphorelay (MSP) and uses three protein species to bring the CK signal up to the primary response genes: (i) transmembrane catalytic receptors with histidine kinase activity, (ii) mobile phosphotransmitters circulating between the cytoplasm and nucleus, and (iii) nuclear transcription factor, namely B-type response regulators. Other proteins (cytokinin response factors (CRFs), pseudophosphotransmitters, A-type response regulators) affect the intensity of the CK signaling through the main transmission pathway (Kieber and Schaller, 2014, 2018).

Receptors are key factors in the perception and transduction of hormonal signals. In the case of CKs, receptors are sensory hybrid histidine kinases largely homologous to bacterial sensory histidine kinases, members of a two-component signal transduction system. Known CK receptors are multidomain proteins located mainly in ER membranes (Caesar *et al.*, 2011; Lomin *et al.*, 2011, 2018; Wulfetange *et al.*, 2011; Daudu *et al.*, 2017; Ding *et al.*, 2017) with an N-terminal hormone-binding sensory module localized in the ER lumen and the central and C-terminal catalytic domains protruding into the cytosol (Steklov *et al.*, 2013; Lomin *et al.*, 2018). Until now, CK receptors have been studied in few vascular plant species, primarily and in most detail in Arabidopsis and maize (Kakimoto, 2003; Yonekura-Sakakibara *et al.*, 2004; Romanov *et al.*, 2006; Lomin *et al.*, 2011, 2012, 2015, 2018; Stolz *et al.*, 2011; Heyl *et al.*, 2012; Steklov *et al.*, 2013; Wang *et al.*, 2014). In recent years, CK receptor studies have been extended to other species including rice (Choi *et al.*, 2012; Ding *et al.*, 2017), *Lotus japonicus* (Held *et al.*, 2014), *Medicago truncatula* (Laffont *et al.*, 2015; Boivin *et al.*, 2016), oilseed rape (Kuderová *et al.*, 2015), *Nicotiana attenuata* (Schäfer *et al.*, 2015), and apple (Daudu *et al.*, 2017). These studies have demonstrated that the CK perception apparatus in some aspects is species-specific. Potato differs from most plant species by its ability to form tubers. This process, sensitive to various cues including CKs, makes the study of CK receptors of potato especially intriguing. So far, to our knowledge, there have been no scientific reports on such studies.

In this paper, we examined potato CK receptors of a homozygous doubled monoploid Phureja (DM1-3 516 R44) whose genome was sequenced several years ago (Potato Genome Sequencing Consortium, 2011). Cloning and expression of receptor-encoding genes were conducted using the commercial autotetraploid potato cv. Désirée. The presence of all necessary MSP elements in potato was demonstrated and the main characteristics of cyclases/histidine kinases associated sensory (CHASE) domain-containing CK receptors were ascertained, primarily their consensus motifs, 3D structure, ligand-binding properties, and the ability to transmit the signal by MSP. In contrast to the Phureja monoploid, distinct alleles for each of the three main forms of receptors were found in the Désirée potato. Expression of CK receptor genes was shown to be organ-specific and affected by sucrose. The results obtained may serve as a framework for new biotechnological approaches in improving potato productivity and stress resistance.

## Materials and methods

### Sequence analysis

Nucleotide/polypeptide sequences of CK receptors and other proteins related to the CK signaling were retrieved from the databases NCBI (http://www.ncbi.nlm.nih.gov), Phytozome 11 (https://phytozome. jgi.doe.gov/pz/portal.html), MSU Rice Genome Annotation Project Release 7 (http://rice.plantbiology.msu.edu/), and congenie.org (http://congenie.org/) using the BLASTP tool and *AHK2* (AT5G35750), *AHK3* (AT1G27320), *AHK4* (AT2G01830), and other CK-related genes of Arabidopsis as templates. Domain structure of proteins was determined with PROSITE (http://prosite.expasy.org/). Transmembrane domains were determined using the MESSA service (http://prodata.swmed.edu/MESSA/MESSA.cgi;Cong and Grishin, 2012). Domain visualization was performed using the MyDomains—Image Creator service (http://prosite.expasy.org/mydomains/).

Phylogenetic analysis was performed using the MEGA6.0 (Tamura *et al.*, 2013). Alignment of nucleotide sequences (coding sequence (CDS), codon mode) was performed with the ClustalW algorithm. The maximum likelihood method was employed for phylogenetic reconstruction. The search for key amino acids in receptor domains by alignment and visualization of protein sequences was carried out in Clustal X2.1 (Larkin *et al.*, 2007) and Jalview (Clamp *et al.*, 2004), respectively.

### Homology modeling

A search of templates for homology modeling was performed with the SWISS-MODEL web-service (https://swissmodel.expasy.org/;Biasini *et al.*, 2014). Modeling of potato (*Solanum tuberosum* L.) protein structures was accomplished in Modeller 9.19 (https://salilab.org/modeller/;Sali and Blundell, 1993) using the *automodel* class for comparative modeling. For each protein, 200 models were built, and the best model was selected according to the Discrete Optimized Protein Energy (DOPE) score (Shen and Sali, 2006) as determined by Modeller. Templates for modeling and respective references (Müller-Dieckmann *et al.*, 1999; Hothorn *et al.*, 2011; Pekárová *et al.*, 2011; Bauer *et al.*, 2013; Mayerhofer *et al.*, 2015; Dubey *et al.*, 2016) are listed in Supplementary Table 1 at *JXB* online. After adding hydrogen atoms, models were energy minimized in USCF Chimera 1.12 (http://www.cgl.ucsf.edu/chimera/;Pettersen *et al.*, 2004) using the AMBER ff14SB force field (Maier *et al.*, 2015) with 300 steps of steepest descent and 300 steps of conjugate gradient optimization; step size was 0.02 Å in both cases. Stereochemical quality of the models was assessed with ProCheck (Laskowski *et al.*, 1993) implemented with the PDBsum Web service (www.ebi.ac.uk/pdbsum), ProSA-web (https://prosa.services.came.sbg.ac.at/prosa.php;Wiederstein and Sippl, 2007) and QMEAN server (https://swissmodel.expasy.org/qmean/help;Benkert *et al.*, 2009). Visualization and superposition of the models were accomplished with UCSF Chimera. All structure models and respective alignments are available in Supplementary Dataset S1.

### Promoter analysis

Promoter regions of Arabidopsis CK receptor genes (*AHK2*, *AHK3*, and *AHK4*) were obtained from the TAIR database (https://www.arabidopsis.org). Identification of promoter regions of CK receptor genes (*StHK2*, *StHK3*, and *StHK4*) of potato was performed using Phytozome 11 and NCBI databases. The DNA sequence of 1000 nt upstream of the gene transcription start was taken as a promoter region. The search for *cis*-regulatory elements in promoters of the genes studied was carried out using the PLACE (http://www.dna.affrc.go.jp/htdocs/PLACE/) and PlantCARE (http://bioinformatics.psb.ugent.be/webtools/plantcare/html/) programs.

### Receptor cloning

Experiments were performed with autotetraploid potato (*Solanum tuberosum* L.) plantlets of the Désirée variety. Plants were propagated by *in vitro* cloning on Murasige–Skoog (MS) agarose medium supplemented with 1.5% sucrose, at 20 °C and 16 h photoperiod in a controlled climate chamber with luminescent white light illumination (Kolachevskaya *et al.*, 2015, 2017). Total RNA was isolated from single potato shoots and treated with RNase-free DNase I (Thermo Fisher Scientific). Reverse transcription was performed with RevertAid ™ according to the manufacturer’s instructions (Thermo Fisher Scientific). Total DNA was isolated from shoots of individual plants using the CTAB method. The resulting cDNA and total DNA were used to amplify genes encoding predicted potato CK receptors with high-precision Phusion High-Fidelity DNA polymerase (Thermo Fisher Scientific). The primer design was performed to amplify the full-length and truncated (sensory modules with flanking transmembrane helices) CDS of the CK receptors according to sequences in NCBI GenBank, namely XM_015303261.1, XM_006352114.2, and XM_006354988.2. Primer sequences are shown in Supplementary Table S2. PCR products were gel purified and cloned, using the PCR Cloning Kit (Thermo Fisher Scientific), into the plasmid pJET1.2/blunt according to the manufacturer’s instructions followed by transformation of *Escherichia coli* strain DH10B (Invitrogen). *StHK4* was amplified using StHK4_truncated primers. The product was inserted into the construction of pB7FWG2-AHK3 instead of *AHK3*. The latter was removed at the *Bcu*I and *Eco*RI restriction sites (Lomin *et al.*, 2015). The nucleotide sequences of the cloned genes were confirmed by DNA sequencing.

*StHK2* and *StHK3* sequences were subcloned into the plasmid pDONR^TM^221 in BP reaction with Gateway® BP Clonase® II Enzyme mix (Thermo Fisher Scientific). Then, using the LR reaction with the LR Clonase® II Plus enzyme (Thermo Fisher Scientific), the cloned sequence was transferred into the expression vector pB7FWG2 (Karimi *et al.*, 2007) where it was fused at the 3′-terminus to the *eGFP* gene. For expression in *E. coli*, *StHK2* and *StHK4* were amplified using primers StHK2_COLD and StHK4_COLD, respectively (Supplementary Table S2). The product was then inserted into the plasmid pCOLD IV (Takara Bio Inc.) at the *Xho*I and *Xba*I restriction sites for *StHK2* and *Sac*I and *Eco*RI restriction sites for *StHK4*, followed by transformation of the *E. coli* DH10B strain.

### Transient expression of receptor genes in tobacco plants

The transient transformation of tobacco (*Nicotiana benthamiana* Domin) leaves was accomplished according to Sparkes *et al.* (2006). Eight-week-old tobacco plants were infiltrated with a mixture of *Agrobacterium tumefaciens* carrying CK receptor genes fused to green fluorescent protein (GFP) and the *A. tumefaciens* helper strain p19 (Voinnet *et al.*, 2003), and the expression of receptor genes was checked after 5–6 d with a fluorescence microscope, Axio Imager Z2 (Carl Zeiss Microscopy GmbH), before leaves were processed further for microsome isolation.

### Plant membrane isolation

All manipulations were done at 4 °C. Tobacco leaves 6 d after infiltration were homogenized in buffer (3 ml per 1 g of fresh weight) containing 100 mM Tris–HCl (pH 8.0), 2 mM Na_2_-EDTA, 50 mM KCl, 1 mM DTT, and 1 mM phenylmethylsulfonyl fluoride. The homogenate was filtered through Miracloth (Calbiochem, San Diego, CA, USA), and the filtrate was centrifuged for 5 min at 5000 *g*. Then supernatant was centrifuged for 40 min at 15000 *g*. The microsome pellet was resuspended in 50 mM KCl–10% glycerol and the microsome suspension was stored at −70 °C.

### Hormone binding assays

Ligand binding studies were performed in PBS as described previously (Romanov *et al.*, 2005; Lomin *et al.*, 2015). Studies of pH influence on hormone binding were performed in 50 mM MES–KOH (pH 5–7) or Tris–HCl (pH 7–9) buffers with 50 mM KCl. *K*_d_ for [^3^H]*trans*-zeatin (tZ) binding to different receptors was determined in saturation assays followed by data analysis in Scatchard plots.

### Assessment of receptor functionality

Plasmids pCOLD IV with *StHK* coding sequences were transferred for the expression into *E. coli* strain KMI001 (Suzuki *et al.*, 2001). In this strain, the histidine kinase receptor→YojN→RcsB→*cps::lacZ* pathway can be activated by external CKs (Takeda *et al.*, 2001). The activation of the signaling pathway was monitored by measuring β-galactosidase activity of *E. coli* cells. Cultivation of clones on Petri dishes containing 40 mM glucose, 40 μg ml^−1^ X-gal, 100 μM isopropyl β-D-1-thiogalactopyranoside (IPTG), 50 μg ml^−1^ ampicillin at 15 °C was performed for 4 d. The individual clones were then streaked onto new Petri dishes containing 40 mM glucose, 40 μg ml^−1^ X-gal, 100 μM IPTG, 50 μg ml^−1^ ampicillin±*trans*-zeatin at a concentration of 0.5 μM. The clones were grown for 3 d at 15 °C. Expression of the *cps::lacZ* construct was evaluated by blue staining of bacterial clones.

### Gene expression analysis

Potato cv. Désirée plants were cultivated under standard *in vitro* conditions at a long (16 h) day for 5–6 weeks on liquid MS medium containing 1.5% or 5% sucrose. For hormone treatment, the medium was replaced with the same one supplemented with *N*^6^-benzyladenine (BA, 1 μM). Tubes were inverted several times to assure uniform plant wetting and then incubated for 1 h under standard conditions. Finally, plant organs (leaves, stems, roots, tubers) were isolated and immediately frozen in liquid nitrogen. Control plants were treated in the same way but without hormone. Total RNA was isolated by the Trizol method (Brenner *et al.*, 2005), and served as the template for cDNA synthesis by reverse transcription (Invitrogen). All RNA samples were treated with RNase-free DNase I. The resulting cDNA was checked for genomic DNA contamination by PCR with primers differentiating cDNA and genomic DNA. The band derived from genomic DNA was absent in the separating gel. Expression of genes encoding predicted proteins of the CK signaling system was determined by qRT-PCR. Potato housekeeping genes *StEF1*α (elongation factor 1-α, AB061263) and *StCYC* (cyclophilin, AF126551) were used as reference genes (Nicot *et al.*, 2005). Sequences of primers for qRT-PCR are shown in Supplementary Table S2.

### Statistical analysis

Statistical analysis was carried out using Student’s *t*-test and *P*<0.05 was considered as statistically significant. In tables and graph, mean values with standard errors are presented.

## Results

### Monoploid Phureja genome analysis

#### Potato has everything necessary for CK signaling via the MSP pathway

The search for protein sequences and encoding genes involved in CK signaling was performed on the basis of the duplicated potato monoploid Phureja genome (Potato Genome Sequencing Consortium, 2011). In general, all potential components of the canonical CK signaling system described in Arabidopsis and other plant species with a sequenced genome (Kieber and Schaller, 2014, 2018) were identified in potato too. Potential CK-related genes found in potato encode homologs of CHASE domain-containing histidine kinases (CHK), phosphotransmitters (HPt), and response regulators of A (RR-A) and B (RR-B) types (Table 1). This indicates the MSP functioning in potato cells for CK signal transduction, involving proteins of a two-component system. In the potato monoploid proteome, three predicted protein-coding sequences, XP_015158747.1, XP_006352176.1 and XP_006355050.1, orthologous to Arabidopsis receptors AHK2, AHK3 and CRE1/AHK4, respectively, were detected. By analogy with the Arabidopsis orthologs, these proteins were annotated in NCBI as StHK2, StHK3, and StHK4. They correspond to mRNA sequences XM_015303261.1, XM_006352114.2, and XM_006354988.2, respectively. Deduced proteins StHK2, StHK3, and StHK4 share 59.35%, 67.75%, and 67.52% sequence similarity with the Arabidopsis orthologs, respectively. The lengths of *StHK2*, *StHK3*, and *StHK4* genes are 5361, 4352, and 3988 bp, respectively, and predicted proteins are 1263, 1032, and 992 aa long, respectively (Table 1).

**Table 1.** Proteins and genes predictably related to CK signaling system of potato

Protein type	**Protein name**	**Gene ID**	**mRNA**	**Protein**	**Protein length (aa**)
**CHK**	**StHK2**	**LOC102591086**	**XM_015303261.1**	**XP_015158747.1**	**1263**
**CHK**	**StHK3**	**LOC102587294**	**XM_006352114.2**	**XP_006352176.1**	**1032**
**CHK**	**StHK4**	**LOC102603756**	**XM_006354988.2**	**XP_006355050.1**	**992**
HPt	StHP1a	LOC102590747	XM_006365209.2	XP_006365271.1	151
			XM_006365208.2	XP_006365270.1	151
			XM_006365207.2	XP_006365269.1	151
HPt	StHP1b	LOC102603297	XM_006352731.2	XP_006352793.1	152
HPt	StHP1c	PGSC0003DMG400028593	PGSC0003DMT400073603	PGSC0003DMT400073603	148
HPt	StHP6	LOC102601463	XM_006364157.2	XP_006364219.1	156
HPt	StHP4a	LOC102589200	XM_015304066.1	XP_015159552.1	112
			XM_006364659.2	XP_006364721.1	136
HPt	StHP4b	LOC102584884	XM_015315420.1	XP_015170906.1	137
RR-B	StRR1a	LOC102578736	XM_006363517.2	XP_006363579.1	675
			XM_006363518.2	XP_006363580.1	675
RR-B	StRR1b	LOC102586468	XM_006345914.1	XP_006345976.1	663
RR-B	StRR1c	LOC102596771	XM_006349891.2	XP_006349953.1	556
RR-B	StRR14	LOC102606335	XM_006354997.1	XP_006355059.1	653
			XM_006354996.1	XP_006355058.1	656
RR-B	StRR11	LOC102593308	XM_006341706.2	XP_006341768.1	581
			XM_006341705.2	XP_006341767.1	581
			XM_015306278.1	XP_015161764.1	481
RR-B	StRR18a	LOC102598455	XM_006343619.2	XP_006343681.1	681
RR-B	StRR18b	LOC102587717	XM_006350015.2	XP_006350077.1	707
ARR19	StRR19	LOC107060895	XM_015309426.1	XP_015164912.1	371
**RR-A**	**StRR4**	**LOC102602758**	**XM_015313344.1**	**XP_015168830.1**	**248**
**RR-A**	**StRR9a**	**LOC102590336**	**XM_006355533.2**	**XP_006355595.1**	**163**
**RR-A**	**StRR9b**	**LOC102588738**	**XM_015314746.1**	**XP_015170232.1**	**214**
			**XM_015314747.1**	**XP_015170233.1**	**211**
**RR-A**	**StRR9c**	**LOC102599826**	**XM_006351210.2**	**XP_006351272.1**	**226**
**RR-A**	**StRR9d**	**LOC102601166**	**XM_006351214.2**	**XP_006351276.1**	**226**
RR-A	StRR8	LOC102588738	XM_015314747.1	XP_015170233.1	211
			XM_015314746.1	XP_015170232.1	214
RR-A	StRR15	LOC102605280	XM_006344933.2	XP_006344995.1	202
RR-A	StRR17	LOC102583233	XM_006357236.2	XP_006357298.1	156
RR-C	StRR22a	LOC107058083	XM_015303399.1	XP_015158885.1	186
RR-C	StRR22b	LOC107058085	XM_015303400.1	XP_015158886.1	184
RR-C	StRR22c	LOC107059982	XM_015307157.1	XP_015162643.1	137
RR-C	StRR22d	LOC102580685	XM_006361561.2	XP_006361623.2	115

Nomenclature of the NCBI database is used, except StHP1c found only in the Phytozome database. Number of RNA entries exceeds that of genes due to alternative splicing. Data corresponding to CK receptor proteins/genes and response regulator type A proteins/genes studied in this work are highlighted in bold.

#### Phylogenetic analysis classified StHKs into three clades

**Fig. 1.**
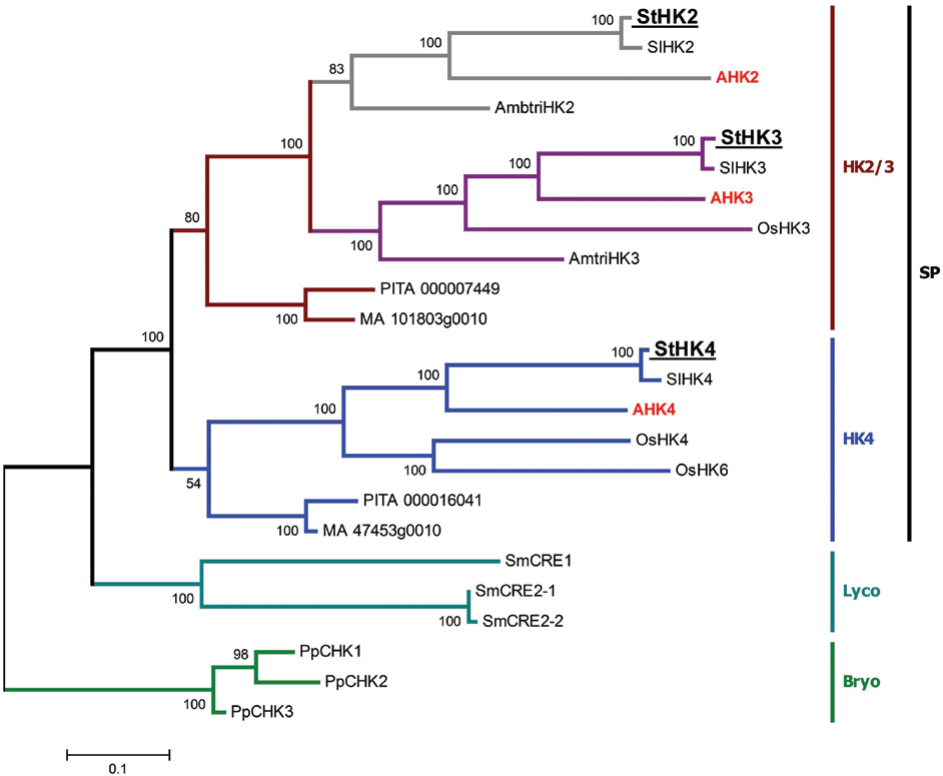
Phylogenetic tree of CK receptors. Species are shown as follows: AHK2-4, Arabidopsis; AmbtriHK2,3, *Amborella trichopoda*; PpCHK1-3, *Physcomitrella patens*; SmCRE1,2-1,2-2, *Selaginella moellendorffii*; MA 101803g0010 and MA 47453g0010, *Picea abies*; OsHK3,4,6, *Oryza sativa*; PITA 000007449 and PITA 000016046, *Pinus taeda*; SlHK2-4, *Solanum lycopersicum*; StHK2-4, *Solanum tuberosum*. SP, seed plants; Lyco, Lycophyta; Bryo, Bryophyta. Parameters of the maximum likelihood algorithm were: phylogeny test—bootstrap method; no. of bootstrap replications, 100; substitutions type, amino acid; model, equal input model; rates among sites, gamma distributed; no. of discrete gamma categories, three; gaps/missing data treatment, complete deletion; ML heuristic method, subtree-pruning, regrafting.

The phylogenetic analysis was performed to compare the conserved and unique features of predicted potato CK receptors with the features of receptors of Arabidopsis, rice, tomato, and other species (Fig. 1). CK receptors of flowering plants can be grouped into three main clades, corresponding to the Arabidopsis AHK2, AHK3, and CRE1/AHK4 receptors (Pils and Heyl, 2009; Lomin *et al.*, 2012; Steklov *et al.*, 2013). Predicted potato and tomato receptors are unequivocally distributed among these three clades. Evolutionarily, they are closer to Arabidopsis than to rice receptors, that was expected since potato, tomato, and Arabidopsis are dicots whereas rice is a monocot.

#### Multiple alignments revealed common and unique features of StHKs

**Fig. 2.**
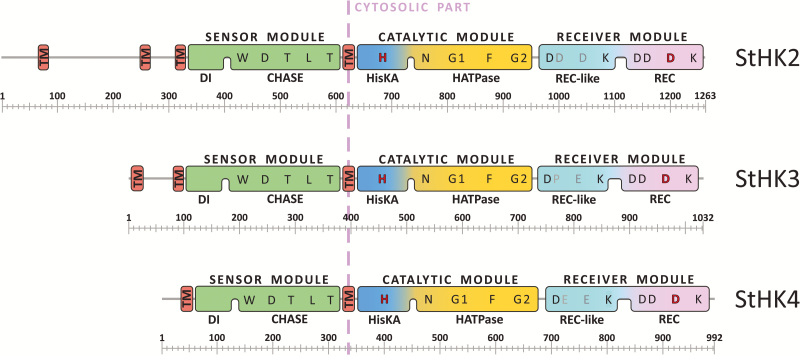
Module/domain structures of the predicted potato CK receptors. Protein domains: CHASE, cyclase/histidine kinases associated sensory domain (Steklov *et al.*, 2013); DI, dimerization interface; HATPase, adenosine triphosphatase domain; HisKA, histidine kinase A domain; Rec, receiver domain; Rec-like, receiver-like domain; TM, transmembrane segment. Conserved amino acids and consensus motifs (N, G1, F, G2) are indicated. According to conventional terminology, the catalytic module consists of a dimerization and histidine phosphotransfer domain (DHpD), and catalytic and ATP-binding domain (CAD) (Mayerhofer *et al.*, 2015; Pekárová *et al.*, 2016). Scales at the bottom of the structures indicate the length in amino acid number.

We investigated the modular architecture of predicted potato CK receptors. The exon–intron structure of the cognate genes as well as occurrence and position of functional domains in the receptor proteins were analysed. Known CK receptors share a common organization, including (from N to C termini) sensory module with CHASE domain, catalytic module with HisKA and ATPase domains, and receiver module with pseudoreceiver and receiver domains (Kakimoto, 2003; Steklov *et al.*, 2013). The sensory module is flanked by predicted transmembrane (TM) α-helices. There is always a single TM-helix C-terminal (downstream) of the module while the number of TM-helices N-terminal (upstream) of the module is variable. The number of upstream TM-helices is usually highest (up to three to four) in AHK2 clade members, lowest (one) in the AHK4 clade and intermediate in the AHK3 clade (Steklov *et al.*, 2013). The domain structure of putative potato receptors fully corresponds to the canonical one (Fig. 2; Supplementary Fig. S1).

**Fig. 3.**
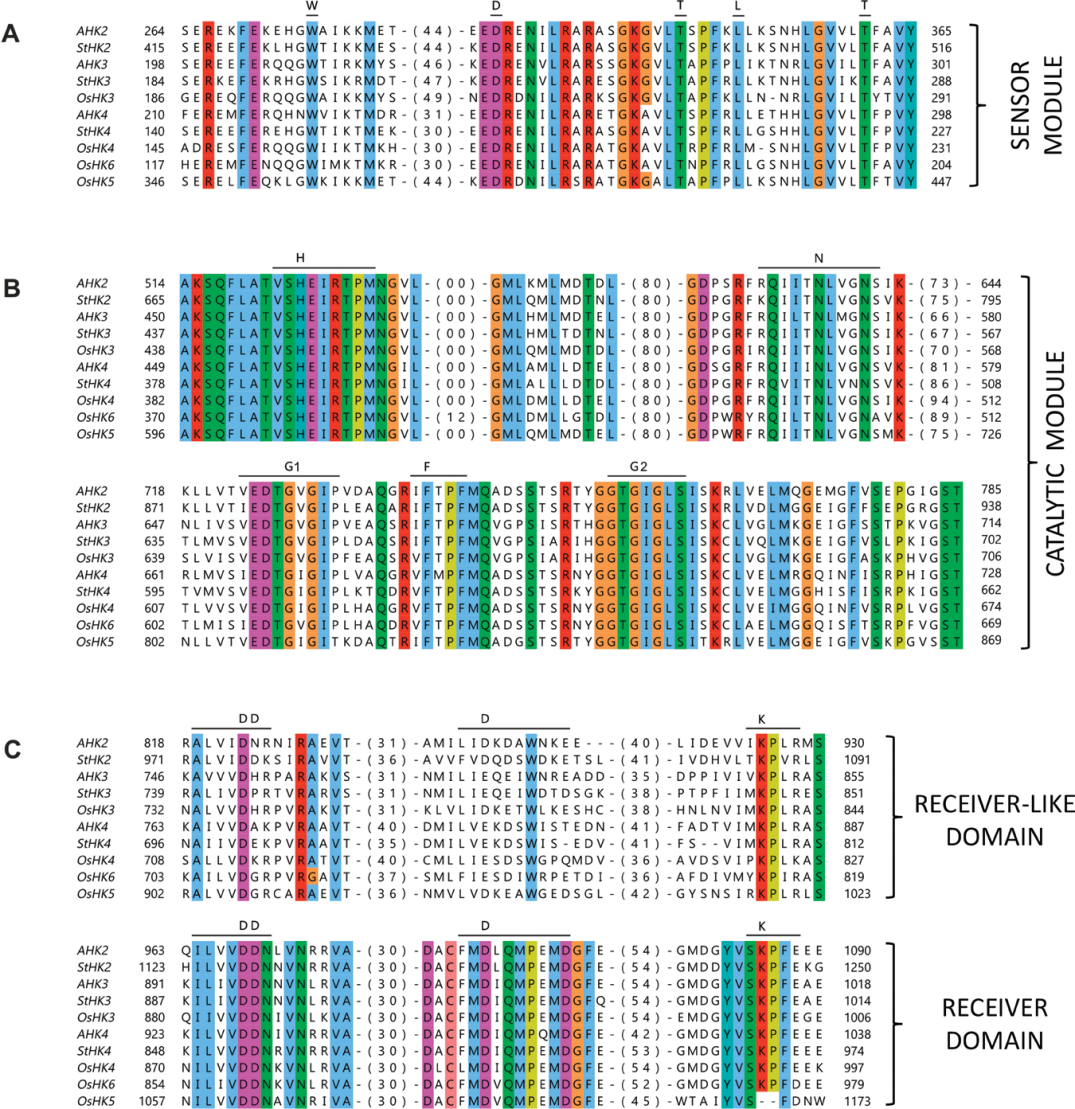
CK receptor sequence alignment. Consensus motifs and conserved amino acids are marked. AHK and OsHK correspond to Arabidopsis and rice proteins, respectively. Number of not shown amino acids is indicated in parentheses.

At the N-termini of potato CK receptors, the number of upstream TM helices is three, two, and one in StHK2, StHK3, and StHK4, respectively. CK receptor genes share similar exon–intron organization. The exon boundaries in the receptor genes of different species coincide in most cases (Supplementary Fig. S1). A multiple alignment of receptor sequences from potato, rice, and Arabidopsis was carried out (Fig. 3). All canonical motifs present in known CK receptors were also found in the potato orthologs. H, N, G1, F, and G2 motifs were identified in the catalytic module, and DD-D-K motifs in the receiver domain of putative potato receptors. Conserved sequences contain phosphorylatable histidine (H) and aspartate (D) residues. StHK2 has a conserved aspartate in its receiver-like domain (Rec-like), similarly to orthologs from Arabidopsis (AHK2), tomato (SlHK2), and rice (OsHK3 and OsHK5). However, the overall DD-D-K-like motifs in Rec-like domains have little in common with the respective sequences in Rec domains (Fig. 3C).

Highly conserved motifs were earlier found in sensory modules and adjacent downstream TM segments of CK receptors (Steklov *et al.*, 2013). These motifs are obviously important for ligand binding and transmembrane signal transfer. In putative potato receptors, these motifs are also present, although with some peculiarities (Supplementary Table S3). In particular, StHK2 has a deviation from the canonical motif in the CHASE domain, where either Glu or Asp is located at position 90, while StHK2 has Gln at this position. StHK3 has a deviation at position 177, strongly conserved in the HK3 clade. This position is occupied by Phe in the canonical motif, and in StHK3 by Leu. In the general histidine kinase motif, either Phe or Tyr is located at position 177. StHK4 is distinguished by positions 83 (Ala→Ser) and 172 (Tyr→Phe) in conserved motifs. Note that counterparts of Gln90, Leu177, and Ser83 are present also in the tomato genome, so these substitutions may be characteristic of the Solanaceae family. Phe172 seems to be unique to potato.

#### StHK functional domains adopt canonical 3D structures

**Fig. 4.**
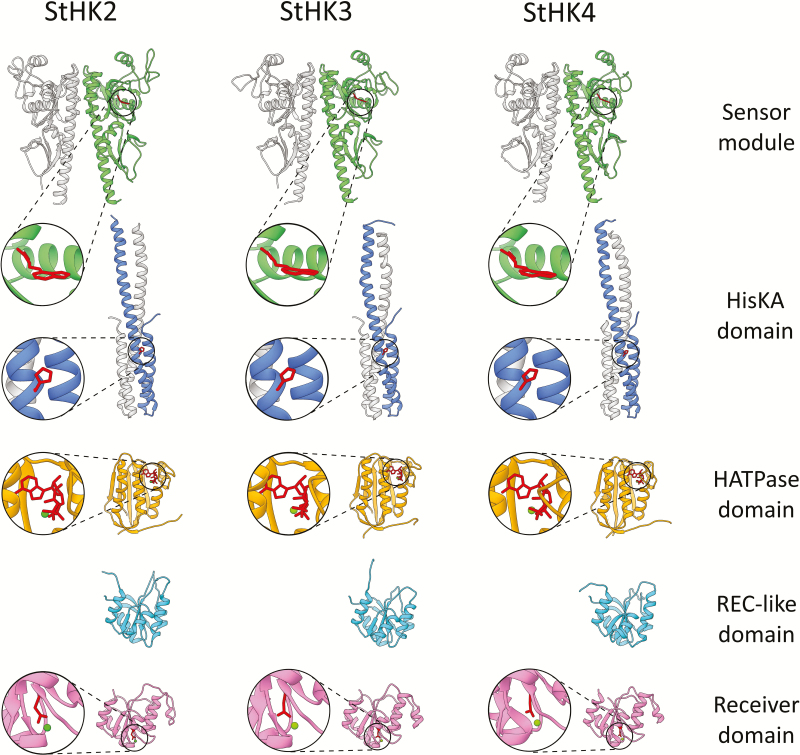
Homology models for predicted potato CK receptor domains. Sensor modules and HisKA domain are presented as dimers where one of subunits is colored grey. Positions of hormone, ATP and phosphoaccepting His/Asp residues are highlighted (red). Green spheres represent Mg^2+^ ions. For additional details, see Supplementary Tables S1 and S4.

We have built homology models of all StHK domains (Fig. 4). High structural similarity of predicted potato receptors with their Arabidopsis orthologs was observed as expected. Key functional regions, such as ligand-binding sites, phosphorylation sites, ATP-binding sites and dimerization interfaces, are particularly conserved. Sensory modules consisting of dimerization, PAS and pseudo-PAS domains (the latter two comprise the CHASE domain) are very similar in Arabidopsis and potato. StHK2 and StHK3 differ from StHK4 by an insertion of 14 and 17 residues, respectively, in the region adjacent to the C-terminus of α3-helix (the first α-helix of the PAS domain) (Supplementary Fig. S2). This insertion apparently does not participate in the hormone recognition site and is unlikely to directly affect the ligand-binding properties of the protein. Similar insertions are also present in AHK2 and AHK3 receptors from Arabidopsis.

The catalytic modules include HisKA domains and H-ATPase domains. HisKA domains are formed by two α-helices and contain a dimerization interface and phosphorylation site (conserved histidine). H-ATPase domains including ATP-binding sites have a sophisticated structure based on parallel/antiparallel β-strands and α-helices. A large insert at the β2–β3 linker (more than 50 residues long) differs CK receptors from bacterial histidine kinases and the H-ATPase domain of the ethylene receptor (Supplementary Fig. S3). This insert is located, however, on the opposite side from the ATP binding site. This structural feature distinguishes not only potato receptors but also CK receptors of other species.

The CKI1 histidine kinase receiver domain (RD), used as the template for CK receptor RD, adopts a fold typical for the REC (or CheY-like) superfamily proteins. It is formed by five α-helices and a β-sheet composed of five parallel β-strands. Two α-helices are located on one side of the β-sheet, and remaining three on the other side. The same fold is characteristic for the model of the Arabidopsis CRE1/AHK4 receptor RD. As distinct from this, an additional small helix is present in the region between the α3 helix and β4 strand in the models of potato and other Arabidopsis receptor (AHK2 and AHK3) RDs, as well as in the CKI2/AHK5 RD crystal structure (Supplementary Fig. S4). A conserved aspartate residue, serving as a phosphate acceptor in RD, is located at the N terminus of the β3 strand (Fig. 4).

Deviations from canonical CHASE motifs in sensory modules of putative potato CK receptors do not seem to alter 3D structures of the modules (Supplementary Fig. S5). An unusual Gln90 resides far from the ligand-binding pocket of StHK2, with side chain directed to the dimerizing interface. Although the unusual Leu177 of StHK3 is localized in the ligand-binding site, its side chain is oriented to the opposite direction. The substitutions in StHK4 seem to be more functional than in other predicted potato receptors. Ser83 and Phe172 are localized in the ligand-binding pocket periphery and their side chains are oriented inwards. Hence, these latter substitutions might somehow influence the ligand specificity of the receptor.

### Experimental studies on autotetraploid potato cv. Désirée

#### Potato cv. Désirée possesses multiple alleles of *StHK* genes

The homozygous doubled monoploid Phureja DM1-3 516 R44 is an artificial form of potato phenotypically differing from commonly known diploid/tetraploid potato varieties (Potato Genome Sequencing Consortium, 2011). Such differences in phenotype are underlain by considerable sequence and structural genome variations between potato haplotypes. Therefore, the results of genome study of monoploid Phureja do not mirror exactly those of more complex genomes of common potato cultivars.

Our experimental study of CK receptors was performed on the autotetraploid potato cv. Désirée, widely used for commercial and scientific purposes (Aksenova *et al.*, 2000; Kolachevskaya *et al.*, 2015). We cloned the putative receptor genes using primers designed according to Phureja gene sequence data. Distinct from the Phureja genome, at least six genes of putative CK receptors were cloned from cDNA of Désirée plants. All these genes share a typical module/domain structure characteristic of hybrid sensor histidine kinases (Figs 2–4). According to their sequence, encoded proteins fall pairwise into three known clades of CK receptors (Table 2; Fig. 1). Thus, each form of CK receptor from potato cv. Désirée consists of at least two close isoforms encoded by natural receptor alleles. Sequencing of cloned genes revealed traits of both similarity and divergence between Phureja and Désirée plants. The nucleotide sequences of HK2-clade members *StHK2a* and *StHK2b* differ from the orthologous Phureja sequence by five and four nucleotides (5 and 4 SNPs), respectively. At the protein level, StHK2a and StHK2b have three and two amino acid substitutions, respectively, relative to Phureja receptor (Table 2; Supplementary Fig. S6).

**Table 2.** Putative CK receptor genes in potato genomes and encoded proteins

Receptor clade	Length of putative CK receptors of potato plants (aa)		SNP/SAP numbers in putative CK receptor genes/proteins of cv. Désirée *vs* var. Phureja		Numbers of Désirée cDNA clones
	**Phureja** ^***a***^	**Désirée** ^***b***^	**DNA bases**	**Amino acids**	
HK2 orthologs	StHK2: 1263	StHK2a: 1263 StHK2b: 1263	5 SNPs 4 SNPs	3 SAPs 2 SAPs	17 17
HK3 orthologs	StHK3: 1032	StHK3a: 1032 StHK3b: 1031	No SNP 23 SNPs, 3 del	No SAP 9 SAPs, 1 del	6 3
HK4 orthologs	StHK4: 992	StHK4a: 992 StHK4b: 991	No SNP 28 SNPs, 3 del	No SAP 12 SAPs, 1 del	1 9

SNP, single nucleotide polymorphism; SAP, single aa polymorphism.

^*a*^Doubled monoploid, method: total genome sequencing.

^*b*^Autotetraploid, method: PCR with cDNA as a template.

Of two cloned genes of the HK3 clade, *StHK3a* is identical to its counterpart in Phureja, whereas *StHK3b* differs by 23 SNPs together with a three-nucleotide deletion. These differences result in the absence of one amino acid and nine amino acid substitutions in StHK3b compared with its Phureja ortholog. Similar data were obtained for the HK4-clade: *StHK4a* was fully identical to that of Phureja whereas *StHK4b* differs by 28 SNPs and a three-nucleotide deletion. Correspondingly, StHK4b differs from its Phureja ortholog, as well as from StHK4a, by deletion of one and substitution of 12 amino acids (Table 2; Supplementary Fig. S6). Analysis of amino acid sequences of the proteins showed that all putative histidine kinases of Désirée potato retain the domains and consensus sequences typical for CK receptors, despite amino acid substitutions (Fig. 2; Supplementary Fig. S6). This indicates that all proteins encoded by the cloned *StHK* genes of tetraploid potato plants can successfully function as CK receptors.

#### StHKs have typical CK-binding properties except StHK3 with distinct ligand specificity

To analyse ligand-binding properties of the receptors, a recently developed plant membrane assay system (Lomin *et al.*, 2015) was used. Predicted potato CK receptor genes were cloned into pB7FWG2 vectors for transient expression in tobacco leaves. In the case of *StHK2* and *StHK4* genes, the full-length cDNA sequences were expressed, but in the case of *StHK3*, expression of the full-length receptor failed for unknown reasons. Instead of full-length receptor, we clond a genomic sequence of the StHK3a sensory module flanked by transmembrane domains. From the transiently transformed tobacco leaves, a microsomal fraction enriched with individual potato receptors was obtained. The binding assays were conducted using this fraction and tritium-labeled CK. In aggregate, we tested four putative receptors belonging to all three clades: StHK2a, StHK3a (sensory module, further designated as StHK3a_SM_), StHK4a, and StHK4b.

**Fig. 5.**
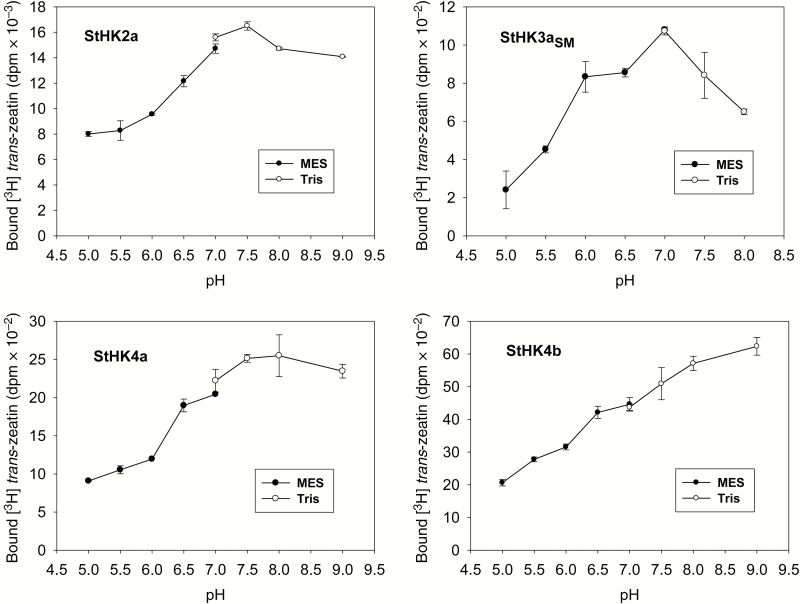
pH dependencies of *trans*-zeatin binding to putative potato CK receptors.

First, we determined the pH-dependence of hormone binding to these receptors within the pH range of 5–9 (Fig. 5). All StHKs exhibited maximal *trans*-zeatin binding at neutral to mildly basic pH: StHK2a at pH 7.5, StHK3a_SM_ at pH 7, StHK4a at pH 7.5–8, and StHK4b at pH 8–9. All StHKs showed a decrease in ligand binding at acid pH: StHK2a and StHK3a_SM_ reduced their binding at pH 5 compared with pH 7 by a factor of 2 and 5, respectively. Ligand binding by StHK4a and StHK4b decreased at pH 5 about 3-fold compared with maximal values. Although the StHK3a was represented in this study only by its sensory module, a control experiment with the full-length StHK2a and its sensory module showed a similar pH-dependence of hormone binding (data not shown). This means that an isolated sensory module is sufficient to determine the pH-dependence of hormone binding by the receptor.

The interaction of a hormone with a receptor is characterized by the equilibrium dissociation constant (*K*_d_) of the ligand–receptor complex. *K*_d_ values were determined by the dose-dependent binding of labeled *trans*-zeatin to StHKs, and the results were processed by the Scatchard method (Supplementary Fig. S7; Lomin and Romanov, 2008). All StHKs demonstrated high affinity for *trans*-zeatin, with similar *K*_d_ values at the nanomolar level (Table 3). The determined *K*_d_ values were close to the values of analogous constants for CK receptors of other species (Lomin *et al.*, 2012, 2015, Kuderová *et al.*, 2015) and were well correlated with concentrations of active CKs *in planta* (Hirose *et al.*, 2008) including potato (Kolachevskaya *et al.*, 2017, 2018).

**Table 3.** The affinity (*K*_d_) of various CKs for putative potato receptors

Cytokinin	Abbreviation	Apparent *K*_d_ (nM)			
		StHK2a	**StHK3a**_**SM**_	**StHK4a**	**StHK4b**
*trans*-Zeatin	tZ	2.6 ± 0.3	4.7 ± 0.6	2.5 ± 0.7	3.0 ± 0.3
*cis*-Zeatin	cZ	102 ± 7	110 ± 39	106 ± 22	129 ± 19
*N* ^6^-Isopentenyladenine	iP	2.4 ± 0.2	5.2 ± 0.8	2.1 ± 0.2	2.5 ± 0.3
Dihydrozeatin	DZ	169 ± 18	21 ± 3	178 ± 37	227 ± 33
*N* ^6^-Benzyladenine	BA	45 ± 3.5	49 ± 7	55 ± 7	63 ± 12
Thidiazuron	TD	1.40 ± 0.04	2.3 ± 0.5	12.6 ± 1.9	17.2 ± 2.5

Different CKs are usually present in the plant: *trans*- and *cis*-zeatins (tZ and cZ), isopentenyladenine (iP), and dihydrozeatin (DZ). In addition to natural CKs, there are many synthetic ones. Receptors exhibit different affinities for these compounds (Lomin *et al.*, 2015; Savelieva *et al.*, 2018). We studied the ligand specificity of putative receptors in competitive experiments where binding of labeled CK was carried out in the presence of various concentrations of certain unlabeled ligands. Based on the obtained competition curves (Supplementary Fig. S8), the apparent *K*_d_ values were determined for each ligand as described (Lomin and Romanov, 2008). We analysed the interaction of StHKs with six CKs, including five natural ones as well as the synthetic urea-type CK thidiazuron (Table 3). The ligand specificity of StHKs showed much in common. All analysed proteins had a high and nearly equal affinity for *trans*-zeatin and isopentenyladenine, apparent *K*_d_ ranging from 2.1 to 5.2 nM. All StHKs bound *cis*-zeatin significantly more weakly, with *K*_d_ over 100 nM. *N*^6^-Benzyladenine (BA) exhibited an intermediate affinity with *K*_d_ ranging from 45 to 63 nM. Regarding the two remaining CKs, StHK proteins showed significant differences. StHK3a_SM_ bound dihydrozeatin with *K*_d_ ~21 nM, much more strongly than other putative potato receptors (*K*_d_ ~170–230 nM). StHK2 and StHK3a_SM_ showed a high affinity for thidiazuron (*K*_d_=1.4 and 2.3 nM, respectively), whereas its affinity for StHK4a and StHK4b was much lower (*K*_d_=12.6 and 17.2 nM, respectively). The CK affinity ranking for StHKs was as follows: StHK2, TD>iP=tZ>BA>cZ>DZ; StHK3, TD>iP=tZ>DZ>BA>cZ; StHK4, iP=tZ>TD>BA>cZ>DZ. The preference profiles of StHK2 and StHK3a_SM_ differ by DZ position, and from (almost identical) StHK4 isoforms by TD position. The greatest differences (in TD and DZ positions) were revealed between StHK3 and StHK4. Although StHK3a was represented in this study only by its sensory module, previous data showed that the sensory module is sufficient to characterize the ligand preference of the full-length receptor (Stolz *et al.*, 2011; Lomin *et al.*, 2015).

#### StHKs are able to trigger signaling via MSP

**Fig. 6.**
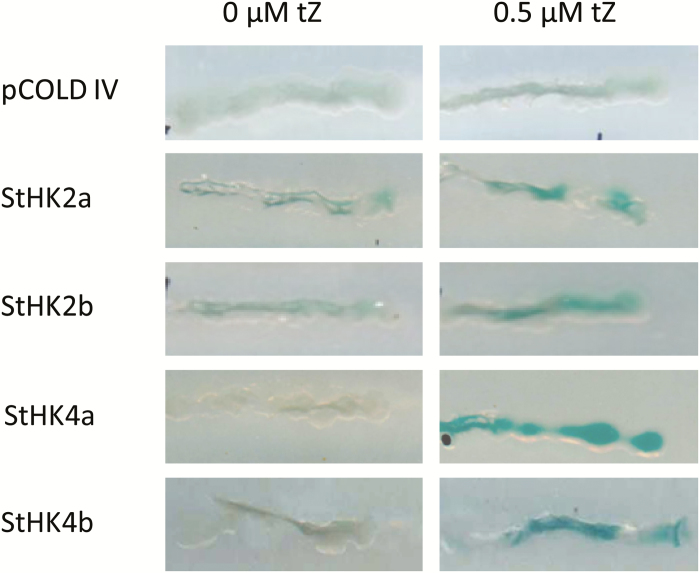
CK receptors of potato feed MSP signaling pathway in ΔrcsC *E. coli* cells.

The ability of the putative potato receptors to trigger CK signaling was tested on *E. coli* ΔrcsC mutant devoid of its own RcsC hybrid histidine kinase and equipped with the *cps:LacZ* construct with the *LacZ* reporter gene driven by *cps* promoter (Suzuki *et al.*, 2001; Takeda *et al.*, 2001). This design allows assessment of the ability of hybrid histidine kinases to initiate signaling over the MSP pathway. Activation of MSP signaling in the bacteria leads to the expression of the reporter galactosidase (LacZ), whose activity is manifested by blueing of clones growing on X-Gal-supplemented medium. We expressed the cloned genes of the putative potato CK receptors in *E. coli* ΔrcsC. In the clones expressing the StHKs, but not in the control clone, blue staining was observed (Fig. 6). The degree of blueing was greatly increased in the presence of CK. This confirms the ability of the cloned potato proteins to transmit the CK signal to the primary response genes via the canonical MSP pathway.

#### StHKs exhibit *in planta* organ-specific expression pattern which has unique properties

To assess the functionality of a gene *in vivo*, it is important to know the level and pattern of its expression in the living organism. We studied the expression of putative CK receptor genes in organs of potato plants grown *in vitro* under conditions favorable for either vegetative growth (1.5% sucrose) or tuber formation (5% sucrose). The mRNA contents of the *StHK2*, *StHK3*, and *StHK4* genes was determined by the qRT-PCR method. For the quantitative comparison of the expression profiles, intra-exon primers were selected for each tested gene (Supplementary Table S1). These primers were complementary to both alleles of the same clade owing to a great similarity of these gene sequences. The relative amounts of putative receptors of distinct clades in potato organs were judged by comparing the levels of transcripts of the cognate genes.

**Fig. 7.**
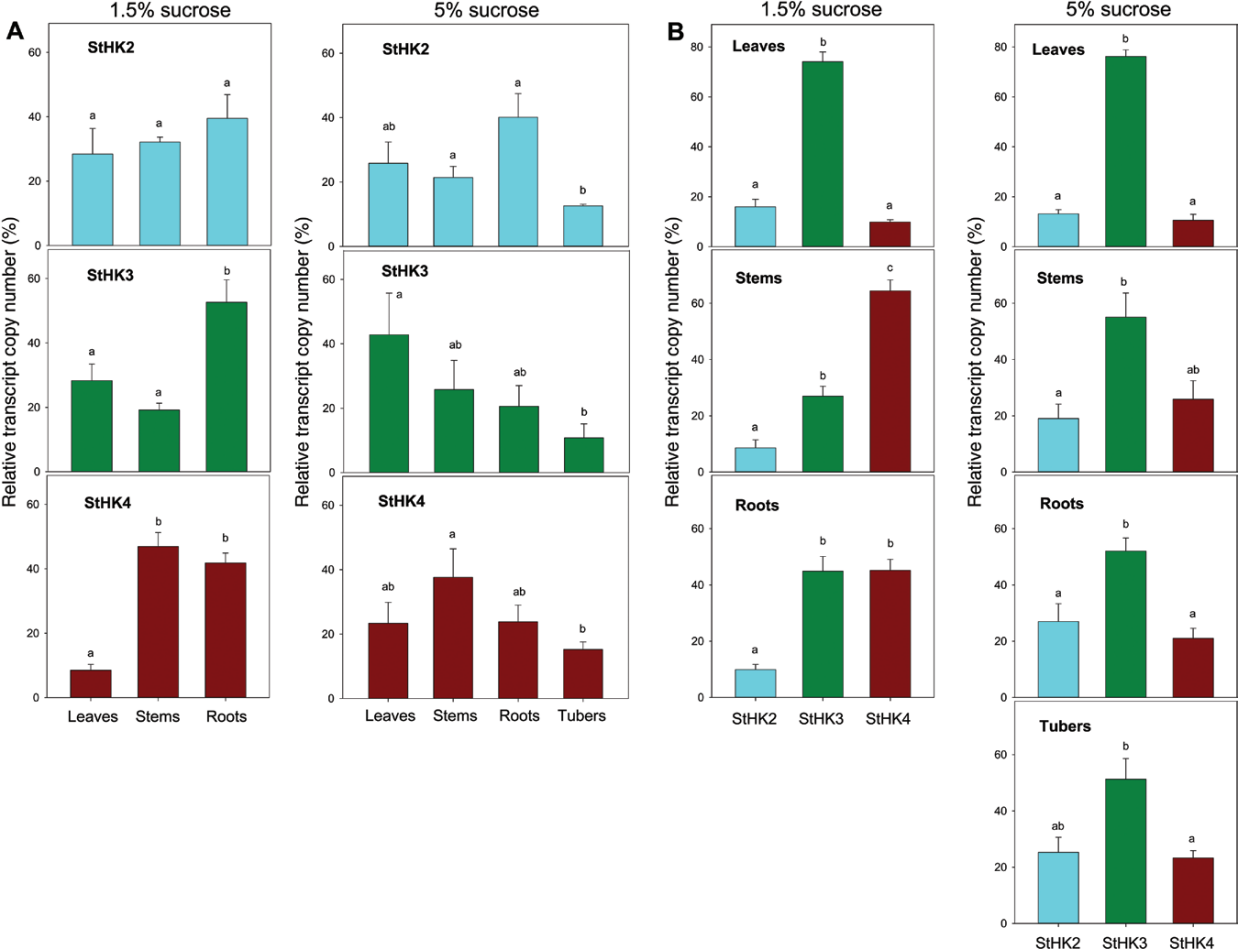
Organ-dependent (A) and clade-dependent (B) patterns of expression of CK receptors in potato plants cultivated on media with different percentage sucrose. Relative transcript copy number is given as percentage of the total transcript amount in each plot, taken as 100%. Different letters (a, b, c) indicate significant differences at *P*<0.05.

Expression levels differed significantly depending on *StHK* group, organ and growth conditions (Fig. 7). Expression patterns were different in plants grown on media with low (1.5%) or high (5%) sucrose content. In the case of 1.5% sucrose medium, the highest expression of the *StHK3* gene was observed in roots, while in the case of 5% sucrose medium, the maximal expression of *StHK3* evidently occurred in leaves. In the low-sucrose grown plants, the *StHK4* gene was much more weakly expressed in leaves than in stems or roots, whereas at the higher sucrose content, level of *StHK4* expression in different organs was more equal. In the *StHK2* group, noticeable organ-specific differences were detected when plants were grown on 5%, but not on 1.5% sucrose. The lowest expression level of all StHK groups was observed, as a rule, in tubers compared with other organs (Fig. 7A).

Within each organ, expression of *StHK3* undoubtedly dominated in leaves, regardless of the sucrose content (Fig. 7B). Expression of *StHK2* and especially *StHK4* genes in leaves was much weaker. In stems grown on 1.5% sucrose, expression of *StHK4* prevailed, while the lowest expression was characteristic of *StHK2* genes. In the roots, expression of *StHK2* genes was relatively weak, whereas the genes of the *StHK3* and *StHK4* clades were expressed actively and in almost equal proportions. A dissimilar pattern of expression was observed in plants grown on 5% sucrose. Here in addition to leaves, in all other organs tested (stems, roots, tubers) the expression of *StHK3* alleles prevailed too, though to a lesser extent than in leaves. Compared with the low-sucrose medium, 5% sucrose increased the relative expression of *StHK2* genes (in stems and roots), while decreasing the level of *StHK4* expression. Thus, unlike Arabidopsis, in potato plants there is evidently no dominance of StHK4 receptors in roots; on the contrary, StHK3 receptors seem to dominate there when cultivating plants on tuber-inductive 5% sucrose. A common feature of potato and Arabidopsis is a very low expression of HK4 orthologs in leaves.

Although the primers used for qRT-PCR did not distinguish closely related isoforms of the CK receptor genes, it is still possible to approximately estimate the relative expression of these alleles. To achieve this goal, data on cDNA clone numbers can be used (Table 2). Within the same clade, relative quantity of cDNA clones harboring a distinct isoform should reflect the relative occurrence of cognate mRNAs. According to the last column of Table 2 referring to the aerial part of potato seedlings, two mRNA isoforms of the HK2 clade were in a 1:1 ratio; among mRNA isoforms of the HK3 clade, *StHK3a* was approximately 2-fold more frequent than *StHK3b*; in the case of the HK4 clade, *StHK4b* was expressed about one order of magnitude more intensively than *StHK4a*.

#### *StHK* promoter activity is hardly affected by CKs, in accordance with low *cis*-element content

**Fig. 8.**
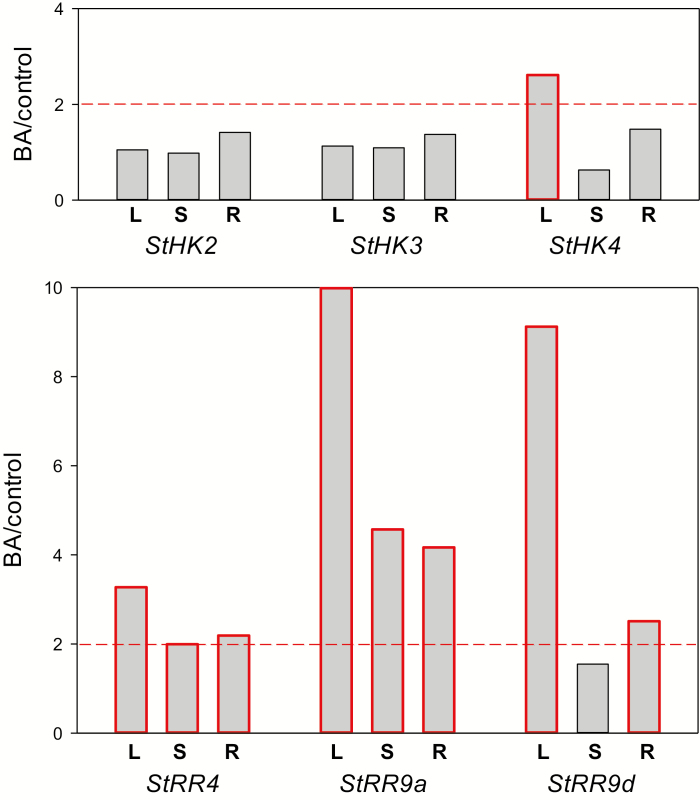
Degrees of transcription induction (BA/control) of CK receptor (top) and response regulator type A (bottom) genes after 1 h treatment of potato plants with 1 μM BA. Plants were grown on MS medium with 1.5% sucrose for 5–6 weeks under standard long day conditions. L, S, and R signify leaves, stems, and roots, respectively. More than 2-fold prevalence of transcripts in BA-treated over control plants is considered as significant induction. Bars corresponding to induced genes are outlined.

Treatment of potato plants with *N*^6^-benzyladenine had a small effect on the expression of the CK receptor genes, and the hormonal impact, when it occurred, was only local and not always reproduced. At 1.5% sucrose, the up-regulation (on average, 2.5-fold) of *StHK4* expression was regularly recorded, but only in leaves (Fig. 8). It can be stated that the CK effect on the expression of potato receptor genes, if any, is mostly limited to *StHK4* and depends on both organ/tissue type and conditions of plant cultivation.

To validate the results of CK treatment experiments, the effect of CK administration on the transcript level of the genes of type A response regulator (*RR*-A) genes was analysed. These genes in other species (Arabidopsis, maize) represent genes of primary response to CK, so it might be expected that in potato too they would be responsive to CK. Indeed, our experiments showed a rapid and reliable increase in the expression of *StRR*-A genes, in contrast to the receptor genes, after plant treatment with BA (Fig. 8). These results prove the reliability of the design and implementation of the experiments and, on the other hand, corroborate the common mode of functioning of the CK signaling system in different plant species.

**Fig. 9.**
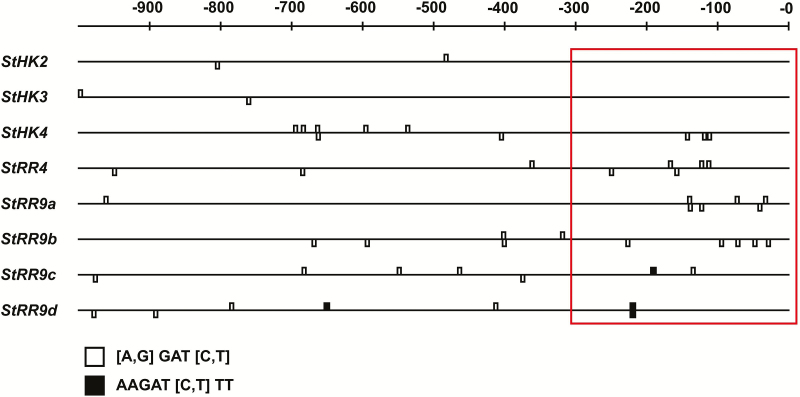
CK-responsive *cis*-regulatory elements in promoters of CK receptor genes (upper part) and response regulators type A genes (lower part) of potato. Elements are shown on both DNA strands. Promoter area proximal to transcription start is boxed.

Analysis of the promoter structures of the studied genes (Fig. 9) was mostly consistent with the gene expression data. Long CK-sensitive *cis*-regulatory elements or blocks of four or more short elements near the transcription start (~300 bp area) were found in promoters of almost all *StRR*-A, but not *StHK*, genes. Among the receptor genes, only *StHK4* has a block of three short CK-sensitive *cis*-elements near the start of transcription. It is possible that this block determines the responsiveness of *StHK4* to CK under certain conditions, as shown in Fig. 8. Though this promoter analysis was accomplished using the genome sequence of var. Phureja, the promoter sequencing from Désirée plants showed an identity of the promoters from these two potato lines.

## Discussion

Plant morphogenesis, in particular tuberization, is based on spatiotemporal cell proliferation and differentiation. One of the main biological effects of CKs is the promotion of cell division (Sakakibara, 2006; Romanov, 2009), and therefore CKs are important participants in morphogenetic processes. Indeed, with regard to potato development, CKs were reported to accelerate and scale up tuber formation (Aksenova *et al.*, 2000; Romanov *et al.*, 2000). In non-potato plants, CKs alone were able to induce the emergence of tuber-like structures (Guivarc’h *et al.*, 2002; Eviatar-Ribak *et al.*, 2013; Frugier *et al.*, 2008; Miri *et al.*, 2016). Apart from the impact on the formation of tubers, CKs are known to regulate overall plant architecture, biomass partitioning, and resistance to biotic and abiotic stress factors (Aksenova *et al.*, 2000; Abelenda and Prat, 2013; Zwack and Rashotte, 2015; Brütting *et al.*, 2017; Thu *et al.*, 2017). All these point to the importance of investigating the CK signaling system in plants, in particular in tuber crops such as potato.

Herein, we present the first results of detailed study of CK receptors from potato plants. Two different potato forms were examined: doubled monoploid Phureja and tetraploid potato of the Désirée variety. Phureja plants possess, like Arabidopsis, three CK receptor orthologs. By contrast, in Désirée plants two allelic forms of each receptor type (StHK2a/b, StHK3a/b, and StHK4a/b) have been found belonging to the three known phylogenetic clades. Our data indicated that this receptor abundance is characteristic of each individual Désirée plant. That implies that the observed differences are not a result of single locus variability, but correspond to genuine paralogs that originated by the polyploidization of the Désirée variety. It is not excluded that the real number of receptor alleles in the potato plant is somewhat higher. Within each group, receptor isomers differ by a few amino acid substitutions, which do not affect most conserved motifs. However, some consensus motifs in the sensory module (Steklov *et al.*, 2013) are distinctive in receptors of potato. The reason for such peculiar properties is not yet clear. Molecular modeling was employed to build models of the structure for all the main domains of potato CK receptors. In general, potato CK receptors share similar domain structure with crystallized hybrid histidine kinases from other species. Note that such a complete characterization of all the main domains of CK receptors is presented for the first time.

The ligand-binding properties of individual potato receptors have been determined: affinity constants for active CKs, pH-dependence of ligand binding, ligand specificity. Two of the studied receptors (StHK3a and StHK4a) are identical in potato cv. Désirée and var. Phureja. All receptors have high affinity for tZ, significantly lower for BA, and relatively low for cZ. StHK3 differs from other potato receptors by relatively high affinity for DZ. The ligand specificity of StHK2 and StHK4 has much in common with that of Arabidopsis orthologs, whereas StHK3 binds iP and BA much more strongly than AHK3, and the affinity of StHK3 for iP and tZ is similar. Thus, the ligand-binding properties of StHK3 differ from those of orthologs in Arabidopsis, maize, and oilseed rape. All receptors bind CK more strongly in the basic (pH 7–9) than in the acidic (pH 5–7) pH range. This is evidence in favor of the intracellular functioning of potato CK receptors (Romanov *et al.*, 2018). The functionality of cloned potato receptors was confirmed by testing their ability to transduce the CK signal via MSP up to the target gene.

The predominant expression of the *StHK3* genes was revealed in leaves, as well as in other organs of plants grown on 5% sucrose, although the degree of dominance of StHK3 was less pronounced in stems, roots, and tubers. When plants were grown on 1.5% sucrose, *StHK4* expression predominated in stems while in roots the expression levels of *StHK3* and *StHK4* were relatively high and nearly equal. In contrast to other species (Romanov, 2009; Lomin *et al.*, 2012), no prevalent expression of HK4 orthologs in roots was found. Exogenous CK had little effect on the expression of CK receptors in potato plants except *StHK4*, which can be rapidly up-regulated in leaves. Analysis of promoter structures showed a correlation between the occurrence of *cis*-regulatory elements and the CK sensitivity of gene expression.

Thus, the totality of our results have left no doubt that the studied StHK proteins are genuine CK receptors in potato. The observed unique structural features refine and broaden our notion of the properties of CK receptors. The revealed peculiarities of the CK perception apparatus in potato might be associated with the ability of this crop to produce tubers. It may be suggested that tuber initiation can be associated with the local/temporary increase in CK signaling in stolon tips. The obtained results create a solid basis for further in-depth study of the role of the CK signaling system in potato ontogenesis and provide new biotechnological tools to optimize hormonal regulation of tuber formation.

## Supplementary data

Supplementary data are available at *JXB* online.

Dataset S1. Structure models and respective alignments of potato CK receptor domains.

Fig. S1. Intron/exon and domain structure of CK receptor orthologs from different plant species.

Fig. S2. Insertions in PAS domains of the sensory modules of StHK2 and StHK3.

Fig. S3. Models of StHK3 HATPase domain.

Fig. S4. Models of receiver domains of StHKs.

Fig. S5. Models of sensory modules of StHKs.

Fig. S6. Alignment of amino acid sequences of CHASE domain-containing histidine kinases from different plant species.

Fig. S7. Scatchard analysis of the interaction between ^3^H-*trans*-zeatin and StHKs.

Fig. S8. Competition of various CKs with ^3^H-*trans*-zeatin for binding to StHKs.

Table S1. Sequence identity of modeled receptor domains and corresponding templates.

Table S2. Primers used in this work.

Table S3. Consensus motifs in CHASE domains of potato and Arabidopsis CK receptors.

Table S4. Main Ramachandran plot parameters of modeled structures.

Supplementary Material

Supplementary Dataset S1

## Abbreviations:

aa: amino acid
BA: *N* ^*6*^-benzyladenine
CHASE: cyclases/histidine kinases associated sensory
CHK: CHASE domain-containing histidine kinases
CK: cytokinin
CRF: cytokinin response factor
cZ: *cis*-zeatin
DZ: dihydrozeatin
GFP: green fluorescent protein
iP: isopentenyladenine
LacZ: galactosidase
MSP: multistep phosphorelay
RR: response regulator
TD: thidiazuron
TM: transmembrane
tZ: *trans*-zeatin.
