# Supplementary material for: Cytokinin perception in potato: new features of canonical players
Source: J Exp Bot. 2018 May 24;69(16):3839–53. doi: 10.1093/jxb/ery199 (PMC6054150; doi:10.1093/jxb/ery199)
Supplement: Supplementary Material [file ery199_suppl_supplementary_material.pdf]

# Cytokinin perception in potato: New features of canonic players

## SUPPLEMENTARY DATA

## Supplementary tables

**Table S1.** Sequence identity of modeled receptor domains and respective templates

| Domain | Template | | Receptor | Identity, % | Reference |
|---|---|---|---|---|---|
| | PDB ID | Protein | | | |
| Sensory module | 3T4L_A | AHK4 | StHK2 | 64.75 | |
| | | | StHK3 | 65.00 | Hothorn *et al.*, 2011 |
| | | | StHK4 | 79.62 | |
| HisKA domain | 4MT8_A | ERS1 | StHK2 | 36.99 | |
| | | | StHK3 | 34.72 | Mayerhofer *et al.*, 2015 |
| | | | StHK4 | 38.20 | |
| ATPase domain | 4PL9_A | ETR1 | StHK2 | 34.94 | |
| | | | StHK3 | 32.74 | Mayerhofer *et al.*, 2015 |
| | | | StHK4 | 31.55 | |
| | 5IDM_A | CckA | StHK2 | 19.88 | |
| | | | StHK3 | 22.42 | Dubey *et al.*, 2016 |
| | | | StHK4 | 22.70 | |
| Receiver domain | 3MMN_A | CKI1 | StHK2 | 48.21 | |
| | | | StHK3 | 43.64 | Pekárová *et al.*, 2011 |
| | | | StHK4 | 48.78 | |
| | 4EUK_A | AHK5/CKI2 | StHK2 | 32.52 | |
| | | | StHK3 | 31.88 | Bauer *et al.*, 2013 |
| | | | StHK4 | 32.37 | |
| Receiver-like domain | 1DCF_A | ETR1 | StHK2 | 25.00 | |
| | | | StHK3 | 15.45 | Müller-Dieckmann *et al.*, 1999 |
| | | | StHK4 | 21.77 | |

**Table S2.** List of primers used in this research

| Primer pair name | Single primer name | Primer sequences (5' – 3') |
|---|---|---|
| StHK2_cloning | StHK2_LP1<br>StHK2_RP3 | GCTTTTCTGCTCTGGGTG<br>TCAACCTGACCCGAAGAAG |
| StHK3_cloning | StHK3_LP1<br>StHK3_RP3 | GGGTTTGGTTTGAAATTGGG<br>GGTATTCTGAGTTGGCTTG |
| StHK4_cloning | StHK4_LP1<br>StHK4_RP3 | ATGGGTGAGAAGATGCAAAGCC<br>CTATTTGTCCGAGTTAGGCTTGG |
| StHK2_sensor module | StHK2_attB1 CHASE<br>StHK2_attB2 CHASE | TACAAAAAAGCAGGCTTGATGGCTCTTGTTATCTTTGTTATTG<br>ACAAGAAAGCTGGGTAAGCATGGAAGATATGACC |
| StHK3_sensor module | StHK3_attB1 CHASE<br>StHK3_attB2 CHASE | TACAAAAAAGCAGGCTTGATGCTTTTGATAGTATG<br>ACAAGAAAGCTGGGTAAAATATTTGCCCTATAAGC |
| StHK2_full length | StHK2_attB1<br>StHK2_attB2 | TACAAAAAAGCAGGCTTGATGAGCTTTTCTGCTCTGGGTG<br>ACAAGAAAGCTGGGTAACCTGACCCGAAGAAG |
| StHK3_full length | StHK3_attB1<br>StHK3_attB2 | TACAAAAAAGCAGGCTTGATGAGTTTGTTTCATGTTATTGGG TTTGGTTTGAAA<br>ACAAGAAAGCTGGGTAGGTATTCTGAGTTGG |
| GATEWAY_stan-dard primers | attB1<br>attB2 | GGGGACAAGTTTGTACAAAAAAGCAGGCT<br>GGGACCACTTTGTACAAGAAAGCTGGGTA |
| StHK4_truncated | StHK4_BcuI<br>StHK4_EcoRI | ACTAGTATGGGTGAGAAGATGCAAAGCC<br>AGGAATTCCAAGTCTCTTCAGATGGTATC |
| StHK2_COLD | StHK2_XhoI<br>StHK2_NheI | ATATCTCGAGATGAGCTTTTCTGCTCTGGG<br>TATGCTAGCTCAACCTGACCCGAAGAAGC |
| StHK4_COLD | StHK4_SacI<br>StHK4_EcoR1a | AAAGAGCTCATGGGTGAGAAGATGCAAAGCC<br>GAATTCCTATTTGTCCGAGTTAGGCTTGG |
| StHK2_qRT PCR(1) | StHK2_FPq1<br>StHK2_RPq1 | ACCATTTGCAGAGACTGGGA<br>GGTCAACAAAAACCACGGCTA |
| StHK3_qRT PCR(1) | StHK3_FPq1<br>StHK3_RPq1 | CACAGCTCCCTTCAGGCTAC<br>TACTCCACCAAGGTACCCGT |
| StHK4_qRT PCR(2) | StHK4_FPq2<br>StHK4_RPq2 | TGCTGAGAGTGGGAAAGCTG<br>GACGTGTAGCCTCAAACCCA |
| StRR4A | StRR4A_FPq1<br>StRR4A_RPq1 | ATCAACACCTTCACCGCCAT<br>TTGAGTCGTCTTGTTGGCGA |
| StRR9A | StRR9A_FPq2<br>StRR9A_RPq2 | CCTCTTATCAAGTTACTGTTGTGGA<br>ACCAGTCATTTCAGGCATGCTA |
| StRR9D | StRR9D_FPq1<br>StRR9D_RPq1 | CCTAGCAACCAACAGGAAGTG<br>TGTTCCTCAGAGATGCAGATTCC |
| EF1_AB061263 | EF1_FP<br>EF_RP | ATTGGAAACGGATATGCTCCA<br>TCCTTACCTGAACGCCTGTCA |
| CYC_AF126551 | CYC_FP<br>CYC_RP | CTCTTCGCCGATACCACTCC<br>TCACACGGTGGAAGGTTGAG |

**Table S3.** Consensus motifs in CHASE domains of potato and Arabidopsis cytokinin receptors

| Type | Amino acid sequence |
|---|---|
| **All CHKs** | xPSAIDQxTF(A)xYTE/ART(A)FERPLT/LSGAVAYAxR/KVx(H)(S)(E)R(E)xFExxxGW(T)IKxMxxx*loop*xxxxxxD/<br>EEYAPV(I)(F)A/SQE/DT(V)(S)H/YxxSxDM(M)SGK/EEDREN(I)LRARA/ES/TGKG/AVLTS/APFR/KLLxS/TN/<br>HH/RLGV(V)LTFA/PVYxxxLPxxx(T)xxxRIxATxG_YLG(G)xF/YDV/IE/PSLV(E)K/NLLxQLAS/GK/NQxIxV(N)<br>VYDxTNx(S)XPI/LxMYGxxxxxx(D)xx(L)x(H)xSxLD/NFGDPxR(K)H(E)Mx |
| **HK2-clade** | xPSAIDQKTF(G)EYTERTxFERPLTSGAVAYALKVxHS(E)R(E)xFE(K)xHGW(T)IKKME(T)(E)*loop*(D)P(A)P(I)DQ<br>EYAPVIFA/SQETV(S)HIVS(I)DMMSG(K)EDR(E)NILRARA(S)GKGVLTSPFKLLKSNHLGVVLTFAVYxxxLPx(D)A<br>T(P)E(Q)R(I)(E)ATxG_YLGASYD(V)PSLV(E)KLL(H)QLASKQTIVV(N)VYDTTN(A)(S)X(P)IxMYGxxxxxxD(T)(G)<br>(L)(L)(H)(I)SN/SLDFGDPxR(K)HEMH |
| StHK2 vs HK2- clade | _PSAIDQKTFEEYTERT_FERPLTSGAVAYALRV_HSERE_FEK_HGWTIKKMESE*loop*DSAPDQDEYAPVIFSQQTV<br>SHIVSIDMMSGKEDRENILRARASGKGVLTSPFKLLKSNHLGVVLTFAVY___LL_YATPVDRINAT_G_YIGASYDVP<br>SLVEKLLHQLASKQTIVVNVYDTTNKF_PI_MYG______DTGLLRVSNLDFGDP_RNHEMH |
| AHK2 vs HK2- clade | _PSAIDQKTFEEYTERT_FERPLTSGAVAYALKV_HSERE_FEK_HGWAIKKMETE*loop*DPAPIQDEYAPVIFAQETVS<br>HIVSVDMMSGEEDRENILRARASGKGVLTSPFKLLKSNHLGVVLTFAVY___LP_DATEEQRVEAT_G_YLGASYDM<br>PSLVEKLLHQLASKQTIVVDVYDTTNTS_LI_MYG______DISEQHISSLDFGDP_RNHEMH |
| **HK3-clade** | xPSAIDQxTF(A)(R)Y(T)(E)RT(A)FERPLTSGAVAYAVRV(L)H(S)ER(E)Q/EFEK/RQQGWT/SI(K)(R)M(D)(T)x*lo*<br>*op*E(P)(S)(P)x(Q)EEYAPVIFAQDTxxHVI/VSxD(M)L(S)G(K)EDRE/DNVLRAR(E)SGKGVLTAPF(R)L(L)(K)TNR<br>LGVI(L)T(F)AVYKxD/ELP(S)NATP(N)ERIQ/EAT(D)G_YLGGV/IFDIE(S)LVEKLL(Q)QLA(S)KQ(T)IxVNVYD(T)T<br>Nx(S)(H)PISMYGS(N)(V)(S)x(D)(G)(L)x(H)VSxLNFGDPxR(K)HEMR/H |
| StHK3 vs HK3-clade | _PSAIDQ_TFASYTERTAFERPLTSGAVAYAVRVLHSERKEFEKRHGWSIKRMDT_*loop*EPSP_QAEYAPVIFAQDT_<br>_HVIS_DMLSGKEDRENVLRARESGKGVLTAPFRLLKTNHLGVIKTFAVYK_DLPSNATPNERIQATDG_YLGGVLDIE<br>SLVEKLLQQLASKQTI_VNVYDMTN_SHPISMYGSNVS_DGL_HVS_LNFGDP_RRHEMR |
| AHK3 vs HK3-clade | _PSAIDQ_TFSEYTDRTSFERPLTSGAVAYAMRVLHSEREEFERQQGWSIRKMYS_*loop*EPSP_QEEYAPVIFAQDT_<br>_HVIS_DMLSGKEDRENVLRARSSGKGVLTAPFPLIKTNRLGVILTFAVYK_DLPSNATPNERIQATNG_YLGGVFDIE<br>SLVEKLLQQLASKQTI_VNVYDMTN_SQPISMYGSNVS_DGL_RVS_LIFGDP_RKHEMR |
| **HK4-clade** | (N)P(S)A(I)DQ(E)TFA(E)YTARTA/SFERPLLSGVAYAxRVxxx(E)R(E)xFEx(Q)(H)GWxIKTMxxxloop(E)(P)S(P)x(<br>R)DEYAPVI(F)(S)Q(E)(T)VSYI/L(E)(S)L/IDMMSGEEDRENILRAR(A)(T)GKAVLTxPFRLL(G)S(H)HLGVVLTFPV<br>Y(K)(S)(K)LPxx(P)(T)x(E)xR(I)xATAG_Y(L)GG(A)FDVESLVENLL(G)(Q)L(A)GNQ(A)I(L)V(N)VYDV/ITNx(S)(D)<br>PLI/VMYGxxxxx(G)(D)xSLxH(E)S(K)LDFGDP(F)RK/RHYM(I) |
| StHK4 vs HK4-clade | NPSAIDQNTFAEYTARTATAFERPLLSGVAYA_RV___ERE_FE_EHGW_IKTM___loopKPSP_RDEYSPVIFSQETVSY<br>IESLDMMSGEEDRENILRARASGKAVLT_PFRLLGSHHLGVVLTFPVYRSKLP__PT_H_RV_ATAG_FLGGAFDVESL<br>VECLLGQLAANHPIIVNVYDVTN_SDPLIMYG_____GD_SL_QVSKLDFGDPFRKHEMI |
| AHK4 vs HK4-clade | NPSAIDQNTFAEYTARTATAFERPLLSGVAYA_RV___ERE_FE_QHNW_IKTM___loopEPSP_RDEYAPVIFSQDSVS<br>YLESLDMMSGEEDRENILRARETGKAVLT_PFRLLETHHLGVVLTFPVYKSSLP__PT_E_RI_ATAG_YLGGAFDVESL<br>VENLLGQLAGNQPIVVHVYDVTN_SDPLIMYG_____AD_SL_HESKLDFGDPFRKHKMI |

CHK means CHASE-domain containing histidine kinase (cytokinin receptor); HK2, HK3 and HK4 mean AHK2-, AHK3- and AHK4-orthologs, respectively. StHKs correspond to potato var. Phureja. In presented motifs (Steklov *et al.*, 2013) bold letters design highly conserved aa positions, regular letters – semiconserved positions, letters in brackets – positions of a prevalent aa, slash-separated letters – positions of any of two amino acids, x – non-conserved position. In the case of individual receptors, non-conserved aa are shown by underlined gaps. Gross deviations in conserved aa sequences of individual receptors are highlighted in red, the same positions in the general motifs and Arabidopsis receptors are marked in green.

**Table S4.** Main Ramachandran plot parameters of modeled structures upon minimization procedures

| Domain | Receptor | Main Ramachandran plot parameters | | | |
|---|---|---|---|---|---|
| | | Most favored regions | Additional allowed regions | Generously allowed regions | Disallowed regions |
| Sensor module (no ligand) | StHK2 | 87.3% | 11.3% | 0.8% | 0.6% |
| | StHK3 | 88.5% | 10.1% | 0.6% | 0.8% |
| | StHK4 | 89.0% | 10.4% | 0.6% | 0.0% |
| HisKA domain | StHK2 | 96.3% | 3.7% | 0.0% | 0.0% |
| | StHK3 | 96.3% | 3.7% | 0.0% | 0.0% |
| | StHK4 | 97.3% | 2.7% | 0.0% | 0.0% |
| HATPase domain | StHK2 | 85.9% | 12.6% | 0.7% | 0.7% |
| | StHK3 | 85.8% | 12.7% | 1.5% | 0.0% |
| | StHK4 | 85.4% | 13.1% | 0.7% | 0.7% |
| Receiver-like domain | StHK2 | 82.0% | 17.3% | 0.0% | 0.8% |
| | StHK3 | 84.1% | 14.3% | 0.0% | 1.6% |
| | StHK4 | 86.0% | 13.2% | 0.8% | 0.0% |
| Receiver domain | StHK2 | 86.5% | 12.7% | 0.0% | 0.8% |
| | StHK3 | 86.6% | 11.0% | 1.6% | 0.8% |
| | StHK4 | 83.9% | 12.1% | 2.4% | 1.6% |

# Supplementary figures

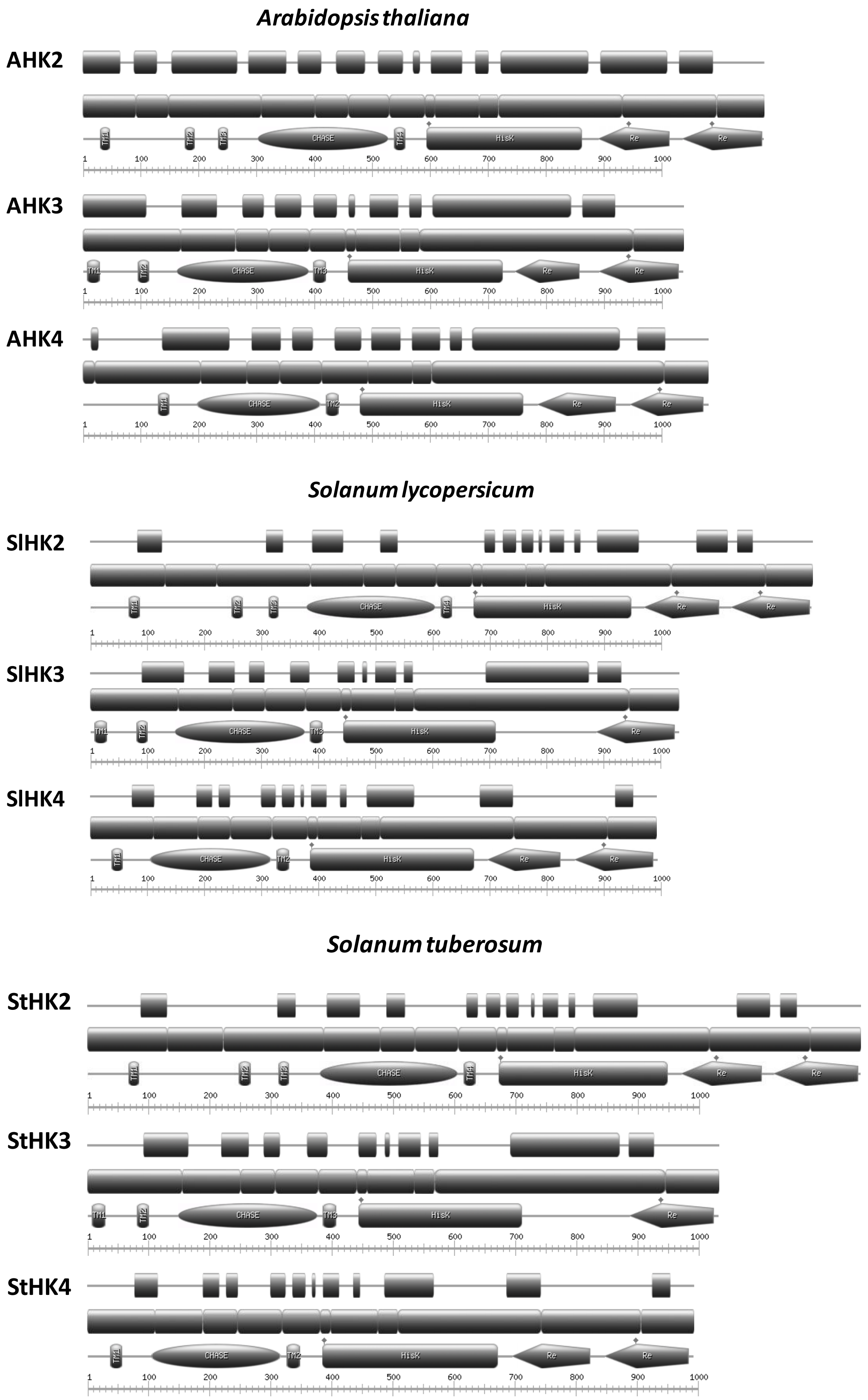


**Fig. S1.** Intron/exon and domain structure of cytokinin receptor orthologs from different plant species. For every receptor, upper line shows scheme of exons (cylinders) and introns (lines) alternation; next line corresponds to RNA/proteins structures upon splicing; third line represents main protein domains; bottom line is the aa scale indicating the protein length.

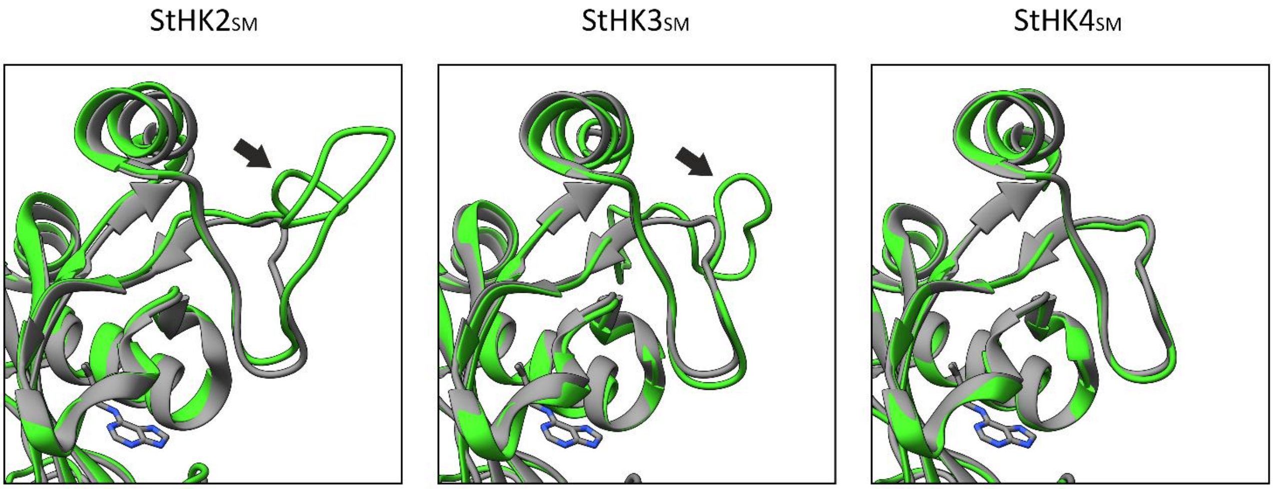


**Fig. S2.** Insertions in PAS domains of the sensory modules of StHK2 and StHK3.
Homology models of sensory modules of *Solanum tuberosum* (var. Phureja) cytokinin receptors (green) are superimposed to crystal structure of Arabidopsis receptor AHK4 (grey) (PDB ID: 3T4L) demonstrating the presence of insertions (indicated by arrows) in StHK2 and StHK3 PAS domains. The ligand position is shown at the bottom of the pictures.

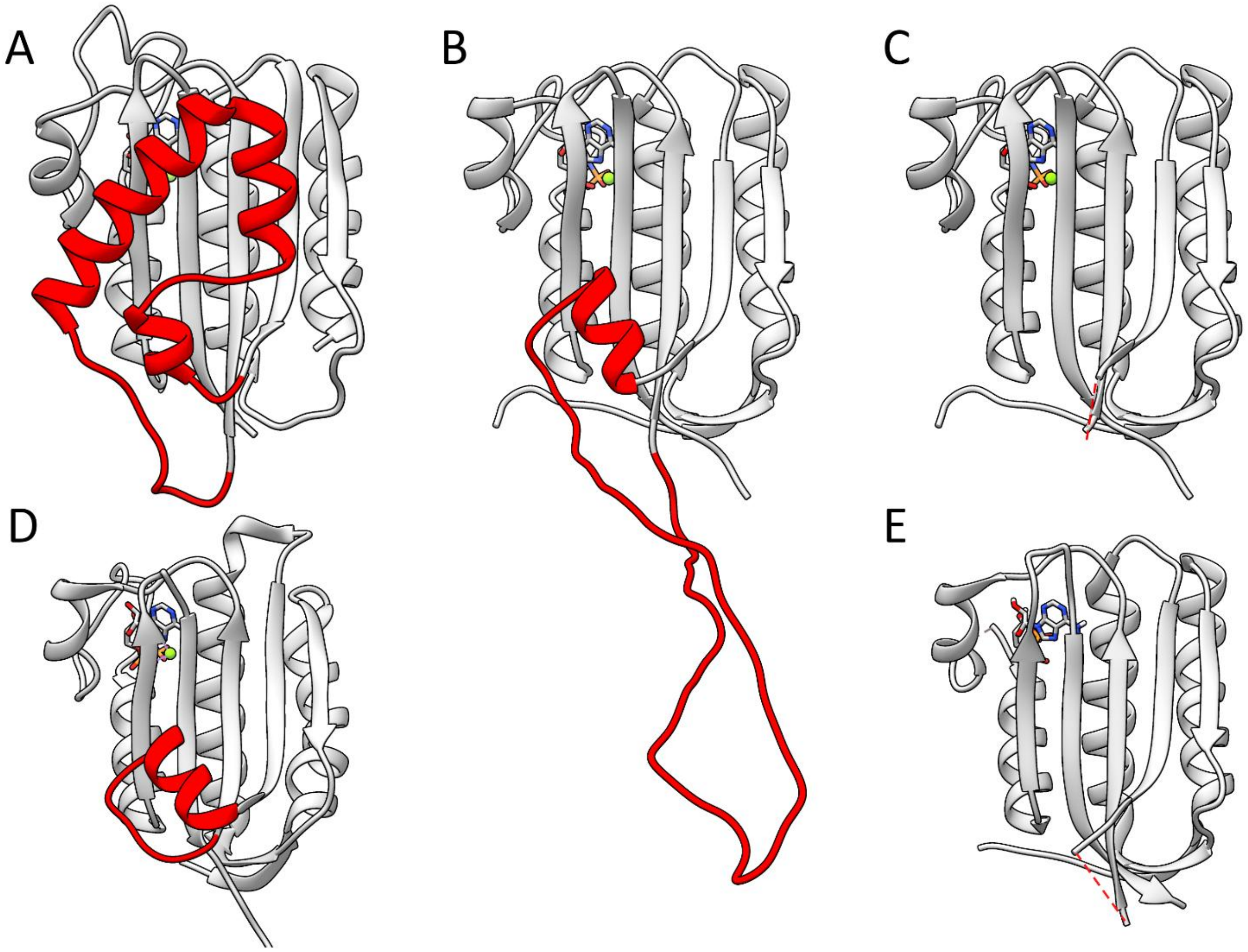


**Fig. S3.** Models of StHK3 HATPase domain built using *de novo* folding with IntFOLD server (A) (Roche *et al*., 2011) and multitemplate homology modelling in Modeller – initial structure (B) and structure with deleted insertion (C) – in comparison with crystal structures of HATPase domain of histidine kinase CckA (D) (PDB ID: 5IDM) and ETR1 (E) (PDB ID: 4PL9) used as templates. Insertion (in comparison with basic HATPase folding) or missing structure (dotted line) corresponding to it are colored red. Small green spheres designate $Mg^{2+}$ ions. It is clearly visible that the insertion is located on the opposite side of the catalytic site.

**Additional reference**

**Roche DB, Buenavista MT, Tetchner SJ, McGuffin LJ.** 2011. The IntFOLD server: an integrated web resource for protein fold recognition, 3D model quality assessment, intrinsic disorder prediction, domain prediction and ligand binding site prediction. Nucleic Acids Research **39**, 171–176.

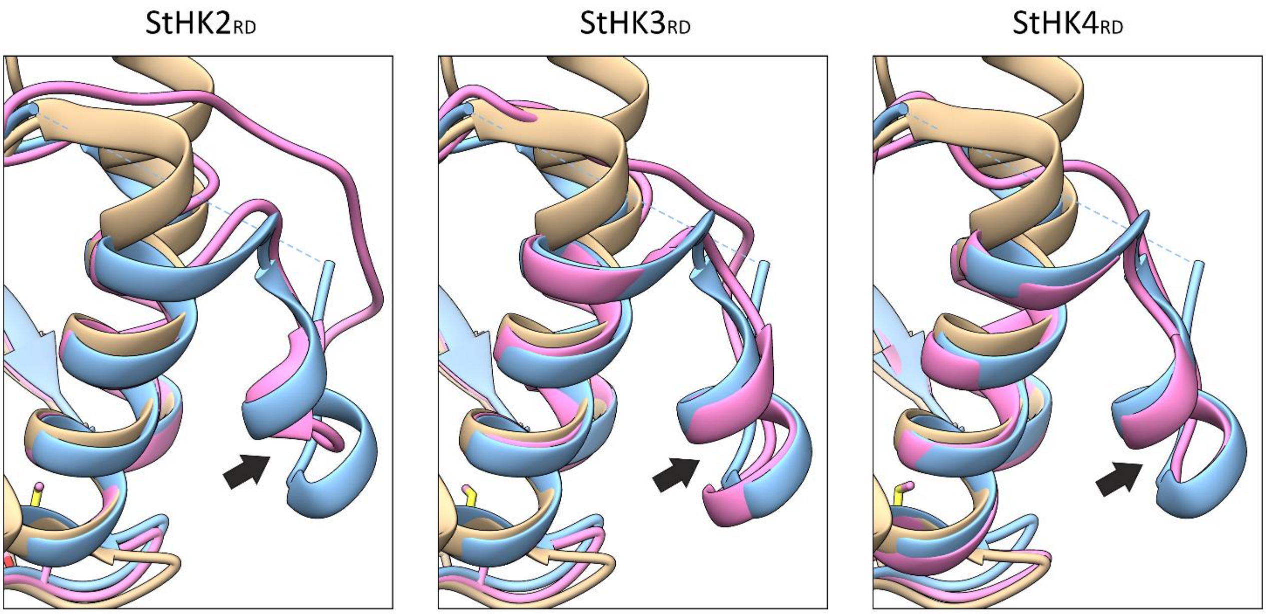


**Fig. S4.** Models of receiver domains of StHKs.

Homology models of StHK receiver domains (magenta) are superimposed to crystal structures of CKI1 (hazel) (PDB ID: 3MMN) and AHK5/CKI2 (blue) (PDB ID: 4EUK, chain A) demonstrating the presence of an insertion and additional 6$^{th}$ α-helix (indicated by arrows) in structures of all StHKs and AHK5/CKI2 in comparison with basic REC-like folding (including CKI1 structure). Missing part of the structure (AHK5/CKI2) is indicated by a dotted line.

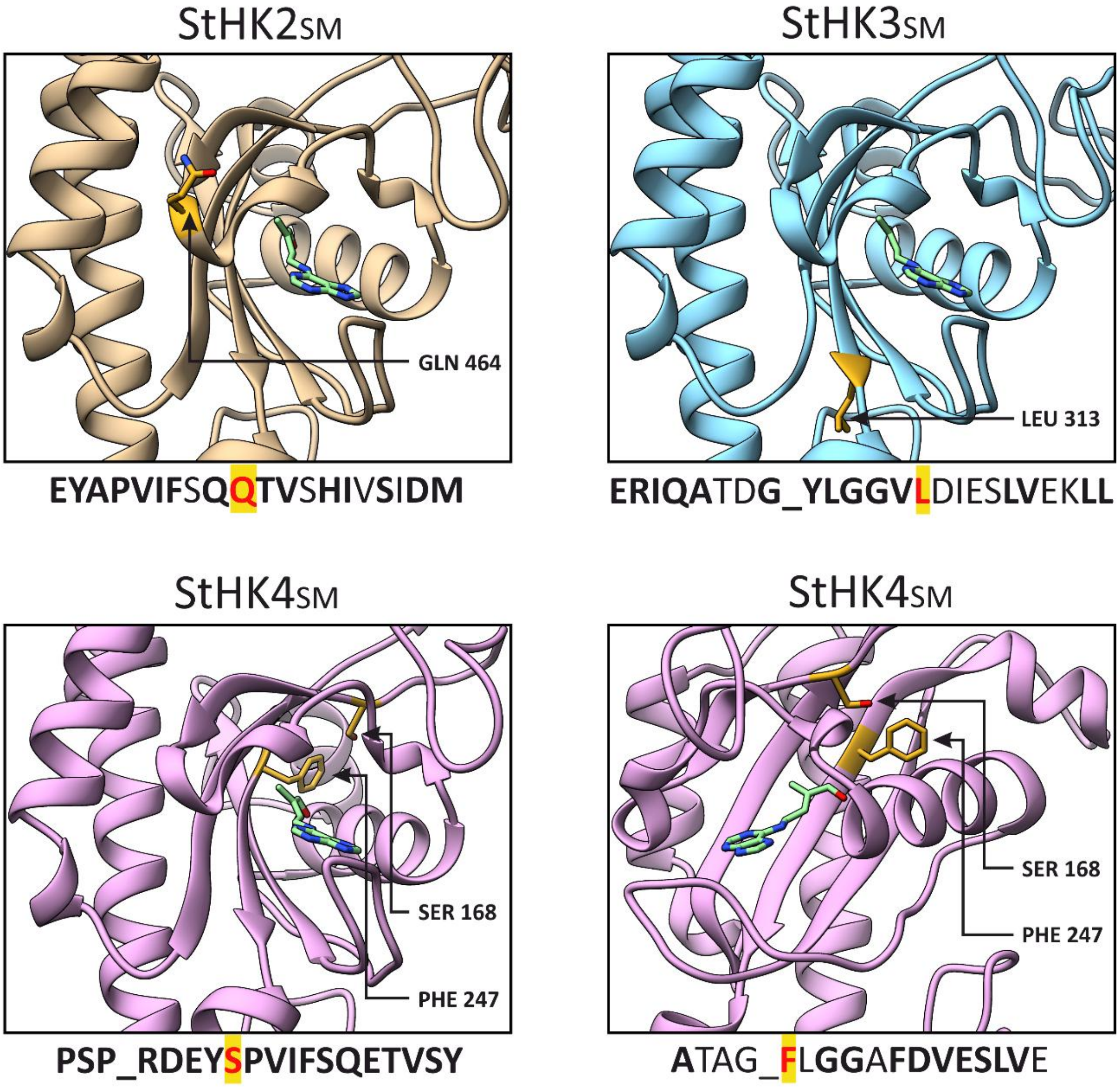


**Fig. S5.** Models of sensory modules of StHKs.

Amino acid residues alterating the canonic motifs are indicated in the models and in respective aa sequence fragments (at the bottom of figures). Sensory module of StHK4 receptor is shown in two orientations. The aa residue numbering is different depending on whether full length proteins or CHASE only (Table S3) are considered. For non-canonical residues, this double numbering is; in StHK2, Gln464/90; in StHK3, Leu 313/177; in StHK4, Ser168/83 and Phe247/172. Non-conserved residues are shown by underlined gaps.

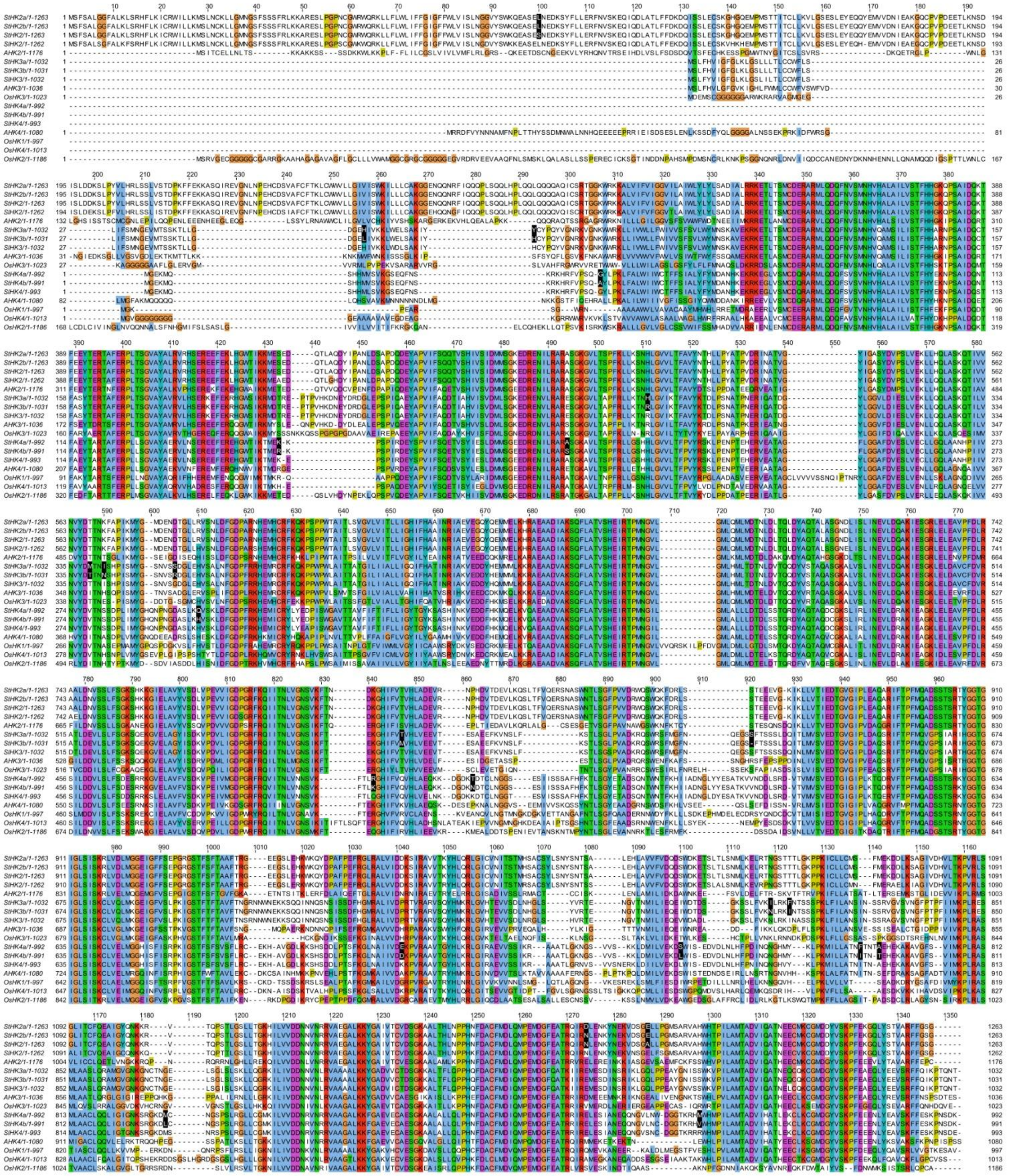

**Fig. S6.** Alignment of aa sequences of CHASE domain-containing histidine kinases from different plant species. Full-length protein sequences from potato var. Phureja (StHK) and Désirée (StHKa/b), tomato (SlHK), Arabidopsis (AHK) and rice (OsHK) are compared. Amino acids differing between Phureja and Désirée sequences are highlighted.

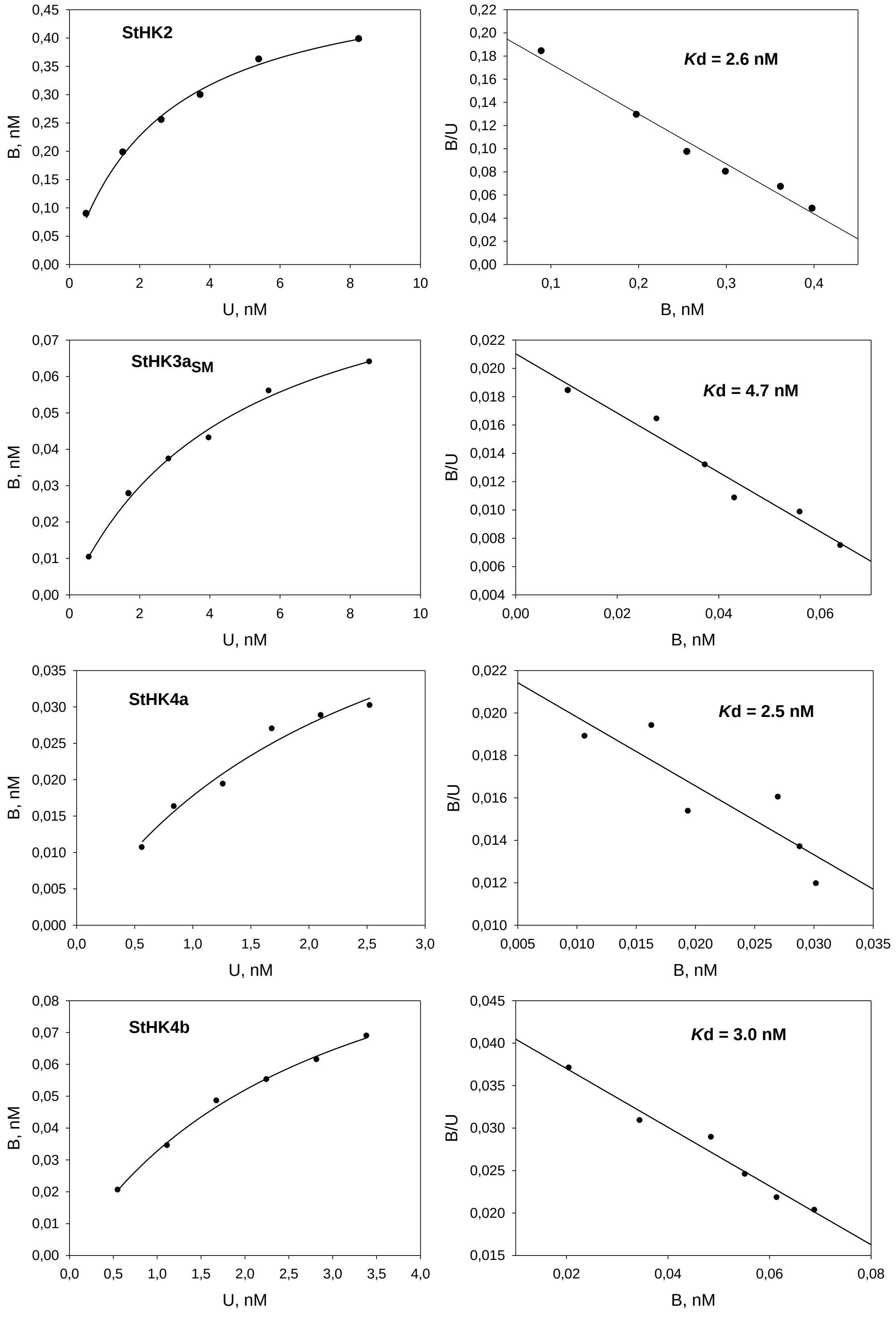


**Fig. S7.** Scatchard analysis of the interaction between $^{3}$H-*trans*-zeatin and StHKs.

On the left, receptor saturation curves; on the right, curve transformation in Scatchard plots. B and U, bound and unbound hormone concentrations, respectively.

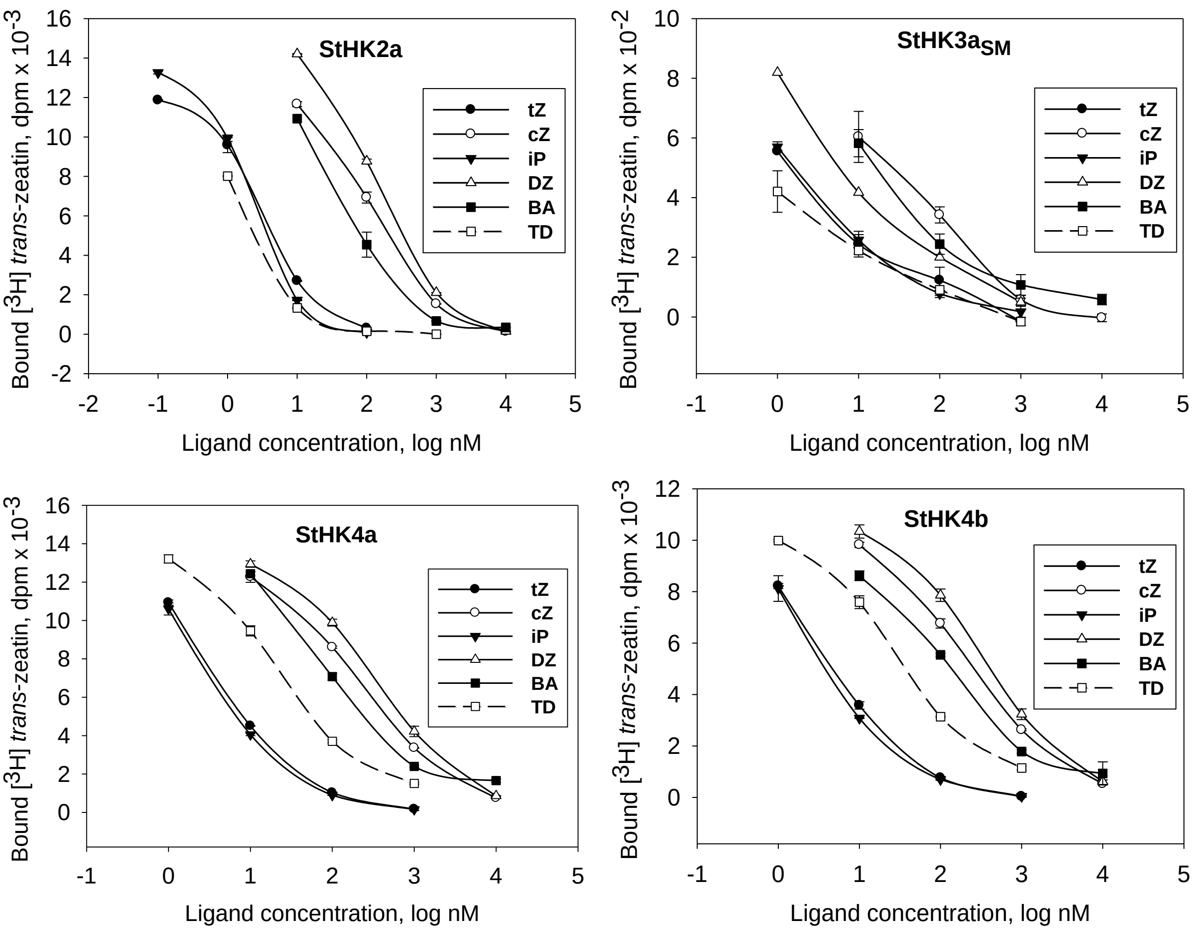


**Fig. S8.** Competition of various cytokinins with $^{3}$H-*trans*-zeatin for binding to StHKs.
Unlabeled hormones are: tZ, *trans*-zeatin; cZ, *cis*-zeatin; iP, isopentenyladenine; DZ, dihydrozeatin; BA, $N^{6}$-benzyladenine; TD, thidiazuron.
